# Cobamide Sharing Is Predicted in the Human Skin Microbiome

**DOI:** 10.1128/msystems.00677-22

**Published:** 2022-08-15

**Authors:** Mary Hannah Swaney, Shelby Sandstrom, Lindsay R. Kalan

**Affiliations:** a Department of Medical Microbiology and Immunology, School of Medicine and Public Health, University of Wisconsin, Madison, Wisconsin, USA; b Department of Bacteriology, College of Agricultural and Life Sciences, University of Wisconsin, Madison, Wisconsin, USA; c Department of Medicine, School of Medicine and Public Health, University of Wisconsin, Madison, Wisconsin, USA; University of California San Diego

**Keywords:** cobamides, ecology, genomics, metagenomics, skin microbiome

## Abstract

The skin microbiome is a key player in human health, with diverse functions ranging from defense against pathogens to education of the immune system. While recent studies have begun to shed light on the valuable role that skin microorganisms have in maintaining the skin barrier, a detailed understanding of the complex interactions that shape healthy skin microbial communities is limited. Cobamides, the vitamin B_12_ class of cofactor, are essential for organisms across the tree of life. Because this vitamin is only produced by a limited fraction of prokaryotes, cobamide sharing is predicted to mediate community dynamics within microbial communities. Here, we provide the first large-scale metagenomic assessment of cobamide biosynthesis and utilization in the skin microbiome. We show that while numerous and diverse taxa across the major bacterial phyla on the skin encode cobamide-dependent enzymes, relatively few species encode *de novo* cobamide biosynthesis. We show that cobamide producers and users are integrated into the network structure of microbial communities across the different microenvironments of the skin and that changes in microbiome community structure and diversity are associated with the abundance of cobamide producers in the *Corynebacterium* genus, for both healthy and diseased skin states. Finally, we find that *de novo* cobamide biosynthesis is enriched only in *Corynebacterium* species associated with hosts, including those prevalent on human skin. We confirm that the cofactor is produced in excess through quantification of cobamide production by human skin-associated species isolated in the laboratory. Taken together, our results reveal the potential for cobamide sharing within skin microbial communities, which we hypothesize mediates microbiome community dynamics and host interactions.

**IMPORTANCE** The skin microbiome is essential for maintaining skin health and function. However, the microbial interactions that dictate microbiome structure, stability, and function are not well understood. Here, we investigate the biosynthesis and use of cobamides, a cofactor needed by many organisms but only produced by select prokaryotes, within the human skin microbiome. We found that while a large proportion of skin taxa encode cobamide-dependent enzymes, only a select few encode *de novo* cobamide biosynthesis. Further, the abundance of cobamide-producing *Corynebacterium* species is associated with skin microbiome diversity and structure, and within this genus, *de novo* biosynthesis is enriched in host-associated species compared to environment-associated species. These findings identify cobamides as a potential mediator of skin microbiome dynamics and skin health.

## INTRODUCTION

The human skin supports a diverse and complex ecosystem of bacterial, fungal, viral, and microeukaryote species, collectively termed the skin microbiome. Highly adapted to live on the skin, these microorganisms form distinct and specialized communities across the skin’s sebaceous, moist, dry, and foot microenvironments. The skin microbiome plays a significant role in human health through contributing to immune system education and homeostasis, protecting against pathogen colonization, and promoting barrier maintenance and repair (1–7). In contrast, loss of microbial diversity on the skin is associated with dermatological diseases such as atopic dermatitis and impaired wound healing (8–10).

The transition from taxonomic characterization of the skin microbiome toward study of the mechanisms driving microbe-microbe and microbe-host interactions has shed light on the truly complex nature of skin microbial communities. Recent work has demonstrated that skin commensals not only take part in synergistic and competitive interactions with other microbes (11–15), but also participate in host interactions that can dictate skin health and function (1, 16–19). While these studies have provided fundamental insight into the roles that certain skin commensals, particularly Staphylococcus species and Cutibacterium acnes, play on the skin, our understanding of the forces that promote stability and mediate overall microbiome structure and diversity on healthy skin is still limited.

Within microbial communities, microorganisms interact at a fundamental level through the competition, acquisition, and sharing of nutrients. Nutritional interdependence, for example, when one member produces a nutrient that is essential for another, has the potential to affect not only individual species dynamics, but also higher-level interactions, dictating microbial community organization, stability, and function. Of particular interest within microbial communities is sharing of the vitamin B_12_ family of cobalt-containing cofactors, cobamides. Here, we use sharing, as previously defined by Sokolovskaya et al. (20), to mean the release of a nutrient or metabolite that is acquired and used by another microorganism.

Cobamides are only synthesized *de novo* by a small fraction of bacteria and archaea, whereas the cofactor is essential for organisms across all domains of life, apart from land plants and fungi (20). They function in the catalysis of diverse enzymatic reactions, ranging from primary and secondary metabolism, including methionine and natural product biosynthesis, to environmentally impactful processes such as methanogenesis and mercury methylation. Across bacteria, an estimated 86% of bacterial species have been found to encode at least one cobamide-dependent enzyme, whereas only 37% of all bacteria are predicted to produce the cofactor *de novo* (21), suggesting that the majority of bacteria must acquire this important molecule externally. A unique feature of cobamides is their chemical diversity and functional specificity, with different microorganisms having distinct cobamide preferences and requirements. As such, numerous mechanisms exist for acquisition and use of preferred cobamide(s), including cobamide-specific gene regulation and selectivity by cobamide-dependent enzymes and transporters (20).

Considering the widespread dependence of cobamides, their limited biosynthesis across bacteria and archaea, and various specificity organism-to-organism, cobamide sharing is hypothesized to be a major driver of microbial community dynamics. Indeed, results from *in silico* studies across diverse environments have supported this hypothesis, providing genomic evidence of cobamide sharing (21–26). Furthermore, *in vitro* and *in vivo* studies of microbial communities, including the human gut microbiome, have demonstrated that cobamide addition modulates community structure, cobamide composition, and expression of cobamide-related genes (20, 27–31). In the skin microbiome, however, the role of cobamides has never before been explored.

In the present study, we generated 268 skin metagenomes from 34 individuals. We then predicted cobamide dependence and biosynthesis within the skin microbiome using these metagenomes and an additional 906 metagenomes generated from 26 individuals across two other independent studies (*n* = 62 individuals and 1,174 total healthy skin metagenomes). We find high concordance between studies showing that phylogenetically diverse skin taxa are predicted to use cobamides, while only a small fraction of species have the genetic potential to produce this important cofactor *de novo.* Modeling of microbial networks shows that cobamide producers, users, and nonusers form positive associations, suggesting cobamide producers are embedded within diverse skin microbial communities. We further found that the relative abundance of cobamide-producing *Corynebacterium* species within a given skin environment is positively correlated with microbiome diversity, a key feature of skin health. Finally, a comparative genomics analysis, including genomes from 71 unique *Corynebacterium* species that represent diverse host and environment niche ranges, shows that *de novo* cobamide biosynthesis is almost exclusively present in genomes of species that associate with hosts, including humans and animals. Taken together, our results highlight the predicted cobamide-sharing capabilities of the skin microbiome, which are likely to be important for mediating community dynamics.

## RESULTS

We performed a cross-study metagenomic analysis of 1,174 skin metagenomes, encompassing samples from 22 distinct skin sites of 3 independent skin microbiome surveys, including Oh et al. (32), Hannigan et al. (33), and data generated from a new cohort for this study (Table 1; Table S1). Although a large number of published skin metagenomes were available as we commenced this study (*n* = 906), they only represented 26 unique individuals sampled at different body sites. To increase the robustness of our analysis, we generated an additional 268 metagenomes from 8 core body sites of 34 individuals, for a total of 62 individuals sampled from three independent studies. For cross-study comparisons, raw sequencing data for all 1,174 samples were processed in parallel using identical methods (see Materials and Methods).

**TABLE 1:** Description of studies

Study	Total no. of skin samples	No. of participants	No. of body sites sampled	Median read count
Present study	268	34	8	17.4 million
Oh et al. (32)	594	12	19	16.9 million
Hannigan et al. (33)	312	16	8	1.2 million

### Cobamide biosynthesis and precursor salvage genes are encoded by select skin taxa.

**FIG 1:**
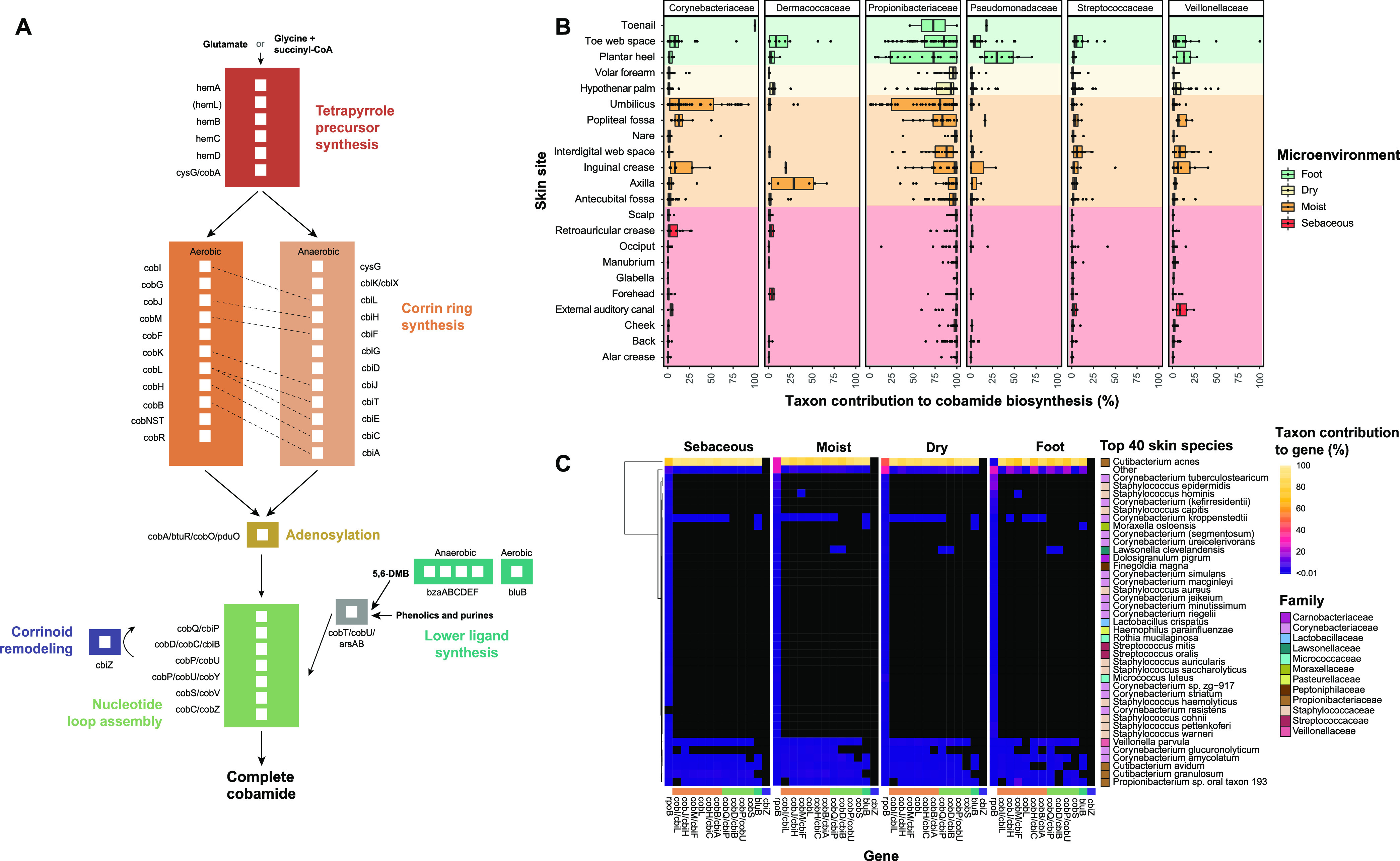
*De novo* cobamide biosynthesis is limited to distinct taxa on the skin. (A) Simplified *de novo* cobamide biosynthesis pathway. Subsections of the pathway are indicated by color, with gene names and white boxes indicating each enzymatic step. Aerobic and anaerobic corrin ring synthesis pathways contain orthologous enzymes that are indicated with dashed lines. *hemL* in parentheses is required for synthesis from glutamate. (B) Taxon contribution reflects the proportion of normalized cobamide biosynthesis gene hits assigned to each taxon out of the total normalized cobamide biosynthesis gene hits within a sample. Normalization was performed by dividing hits to each gene by its profile HMM length. Taxon contributions are shown for the top 6 taxa, grouped by skin site. Color indicates microenvironment classification. (C) The top 40 most abundant bacterial species within the data set were determined by totaling the hits to single-copy gene *rpoB* for each species. The remaining species were grouped into “Other.” Species names that are not yet validly published under the International Code of Nomenclature of Prokaryotes (ICNP) are indicated in parentheses. Individual values in the heatmap represent the number of hits assigned to the species for a particular cobamide biosynthesis gene divided by the total number of hits to the gene. Gene hits were normalized by profile HMM length and sequencing depth prior to calculation. Black squares represent taxonomic abundance from 0 to 0.01%. The colored bar above cobamide biosynthesis genes indicates pathway subsection in panel A.

The *de novo* cobamide biosynthesis pathway is well characterized and highly complex, consisting of at least 25 enzymatic steps that can be divided into subsections, including tetrapyrrole precursor synthesis, aerobic or anaerobic corrin ring synthesis, nucleotide loop synthesis, and lower ligand synthesis (Fig. 1A). To determine if cobamide biosynthesis occurs within the skin microbiome, we used HMMER (34) to search 1,174 skin metagenomes for sequencing reads (translated into protein sequences) that aligned to profile HMMs for 11 proteins from the *de novo* cobamide biosynthesis pathway. Subsequently, the metagenomic reads aligning to the HMMs were extracted and taxonomically classified using a kmer-based approach and custom database with Kraken 2 followed by Bayesian estimation of abundance with Bracken (35, 36).

First, to assess the sensitivity and accuracy of HMM metagenomic read alignment and to determine accuracy of taxonomic classification of the reads using Kraken 2 and Bracken, we analyzed 2 mock community samples, a staggered 20-species community and an even 6-species community, using the same methods applied to the skin metagenomes. We found that our methods were able to accurately detect reads mapping to cobamide-related HMMs from species present even below 0.2% relative abundance in the mock community (Fig. S1 in the supplemental material). Furthermore, predicted *de novo* producers present at low abundance in the mock community, including Cutibacterium acnes, Clostridium beijerinckii, and Pseudomonas aeruginosa (0.18%, 1.8%, and 1.8% relative abundance, respectively), were accurately identified, based on the detection and classification of reads mapping to a majority of cobamide biosynthesis gene markers for these species. We determined that the false-positive rate of hits assigned to taxa not present in the mock community samples was an average of 0.093% across the two samples, equating to approximately 93 false positives per 100,000 hits. Additional steps to reduce false positives can be found in Materials and Methods.

10.1128/msystems.00677-22.1 FIG S1 Analysis of mock skin microbial communities. (A and C) A 6-strain even community (40.8 million reads) (A) and 20-strain staggered community (28.8 million reads) (C) were analyzed with the described HMM and Kraken2/Bracken methods, with singleton reads filtered out for visualization purposes. The total normalized hits for cobamide-dependent enzymes, cobamide transport protein *btuB*, and SCG *rpoB* are shown (totals hits normalized to profile HMM length), with the taxonomic abundance of the hits expanded as relative proportions above. The expected relative abundance of the mock communities is indicated to the left, and taxa included in the communities are bolded. (B and D) To demonstrate sensitivity of the described methods, complete genomes for the mock community members from the 6-strain even community (B) and 20-strain staggered community (D) were scanned for each of the indicated genes with KOFamScan or manually by searching the ATCC annotated genome. Presence of the gene within the genome is indicated with an asterisk, and presence of at least 1 or more reads that mapped to the given HMM and were taxonomically classified to each mock species is indicated with a black square. Expected abundance of each species in the mock communities is listed. Download FIG S1, PDF file, 0.4 MB. Copyright © 2022 Swaney et al. 2022 Swaney et al. https://creativecommons.org/licenses/by/4.0/ This content is distributed under the terms of the Creative Commons Attribution 4.0 International license.

We found that samples from sebaceous sites harbored the overall highest median number of hits to cobamide biosynthesis genes, followed by dry, moist, and foot samples (Fig. S2A). The top taxa encoding for biosynthetic genes in descending order were *Propionibacteriaceae*, *Corynebacteriaceae*, *Veillonellaceae*, *Streptococcaceae*, *Dermacoccaceae*, and *Pseudomonadaceae* (Fig. 1B). For a finer taxonomic resolution of the species contributing to *de novo* cobamide biosynthesis on the skin, we determined the taxonomic frequency profiles of 11 core cobamide biosynthesis genes for the top 40 abundant species within the data set. Species encoding the full or nearly complete suite of cobamide biosynthesis markers was consistent across microenvironments, with Cutibacterium acnes being the dominant contributing species (Fig. 1C). Other species contributing to most or all of the biosynthesis marker genes include Corynebacterium amycolatum, Corynebacterium kroppenstedtii, Corynebacterium glucuronolyticum, Veillonella parvula, Cutibacterium granulosum, and *Propionibacterium* sp. oral taxon 193. Several low abundance skin taxa were predicted to encode the full suite of cobamide biosynthesis markers (grouped into the “Other” category), demonstrating that cobamide biosynthesis is encoded by both dominant and rare taxa. In marine and soil environments, archaeal species have been identified as significant cobamide producers (25, 37). However, we detected no cobamide biosynthesis or *rpoB* reads assigned to Archaea after filtering rare and singleton hits (see Materials and Methods), which was performed to emphasize taxa that are conserved across individuals. This suggests that specific archaeal species are not core cobamide producers on the skin, supporting evidence that suggests archaea are rare and uncommon members in the skin microbiome (38).

10.1128/msystems.00677-22.2 FIG S2 (A) The total sum of reads mapping to cobamide biosynthesis genes, cobamide-dependent genes, or single-copy conserved gene *rpoB* within each sample is shown. (B) The top 20 most abundant bacterial families within the dataset were determined by totaling the hits to *rpoB* for each family. The remaining families were grouped into “Other.” Individual values in the heatmap represent the number of hits assigned to the family for a particular cobamide biosynthesis gene divided by the total number of hits to the gene. Gene hits were normalized by profile HMM length and sequencing depth prior to calculation. Black squares represent taxonomic abundance from 0 to 0.01%. (C) The number of unique species encoding *rpoB* or 11 cobamide-dependent enzymes is shown. The number of unique *de novo* cobamide producers was determined by considering species with reads encoding at least 5 of the 10 cobamide biosynthesis gene markers. Download FIG S2, PDF file, 0.7 MB. Copyright © 2022 Swaney et al. 2022 Swaney et al. https://creativecommons.org/licenses/by/4.0/ This content is distributed under the terms of the Creative Commons Attribution 4.0 International license.

Taxa such as *Moraxellaceae* and *Xanthomonadaceae* encode a limited set of cobamide biosynthesis genes, including *cobQ/cbiP*, *cobD/cbiB*, *cobP/cobU*, and *cobS* (Fig. S2B), which can function in cobamide precursor salvage (39, 40). This suggests that cobamide synthesis through precursor salvaging is also occurring on the skin. Furthermore, cobamides are grouped into three classes based on their structurally distinct lower ligand: benzimidazolyl, purinyl, and phenolyl cobamides (20). Most predicted cobamide producers identified in this analysis likely synthesize benzimidazolyl cobamides because they encode *bluB*, the gene responsible for the aerobic synthesis of lower ligand 5,6-dimethylbenzimidazole (DMB) (Fig. 1C; Fig. S2B) (41–43). However, in species such as *V. parvula*, *C. granulosum*, and *C. glucuronolyticum*, *bluB* is absent, suggesting that these species may produce nonbenzimidazolyl cobamides on the skin. In support of this finding, *V. parvula* is a known producer of phenolyl cobamides (44, 45). Overall, our results demonstrate that select taxa within the skin microbiome have the genetic potential to produce chemically diverse cobamides through *de novo* biosynthesis or precursor salvage and that *de novo* biosynthesis is restricted to a limited number of skin species.

### Phylogenetically diverse skin taxa encode for cobamide-dependent enzymes.

**FIG 2:**
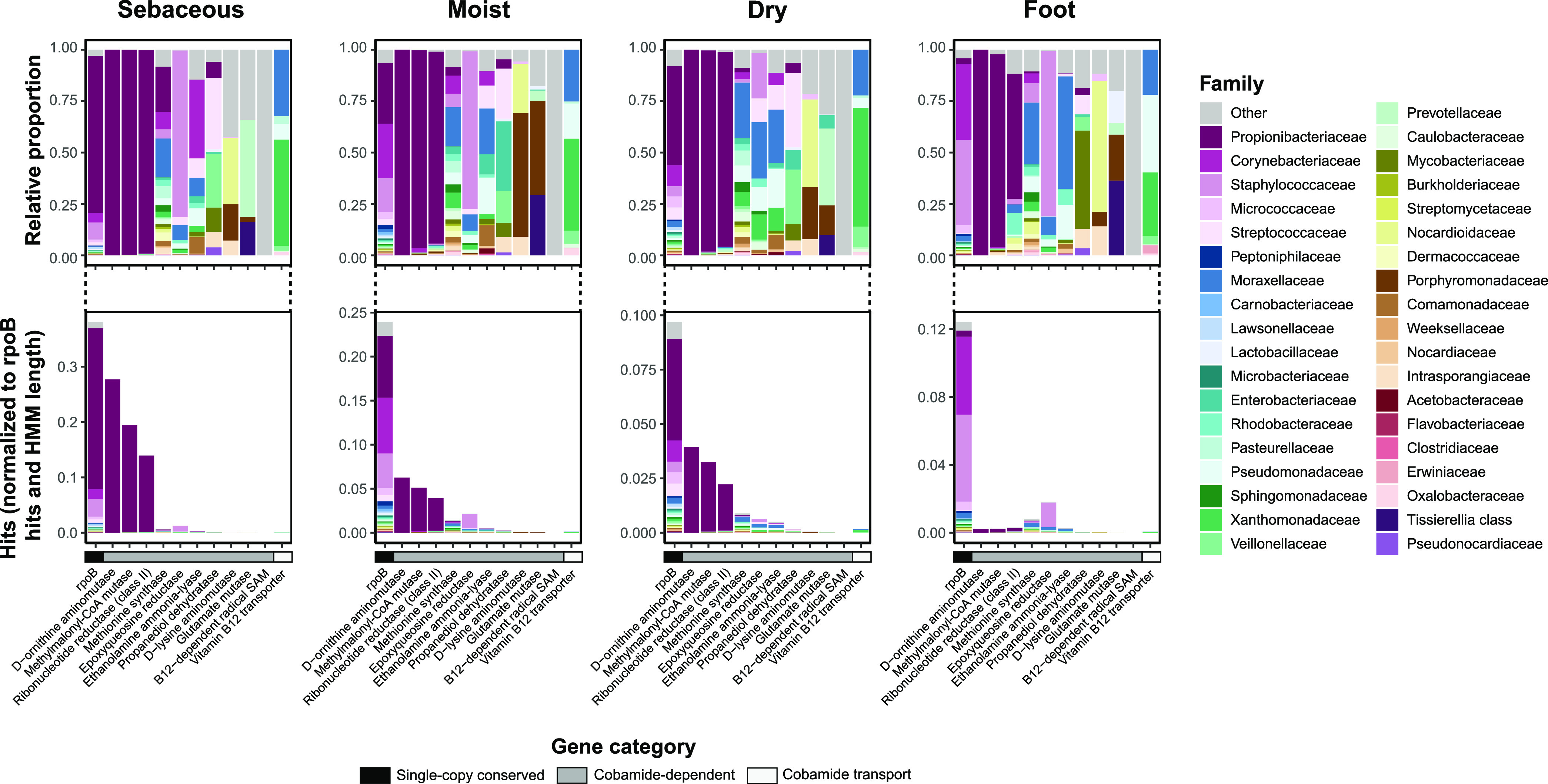
Phylogenetically diverse skin bacteria encode cobamide-dependent enzymes and transporters. The total normalized hits for cobamide-dependent enzymes, cobamide transport protein *btuB*, and single-copy conserved gene *rpoB* are shown (totals hits normalized to profile HMM length and sequence depth), with the taxonomic abundance of the hits expanded as relative proportions above. Hits to distinct B12-dependent radical SAM proteins are grouped together as “B12-dep radical SAM.”

Although relatively few species within the skin microbiome synthesize cobamides *de novo*, we predict that a larger proportion use cobamides. We determined the prevalence of the cobamide transport protein btuB and 19 enzymes that carry out diverse cobamide-dependent reactions. The median number of cobamide-dependent gene hits across samples varied by microenvironment (Fig. S2A). Across the sebaceous, moist, and dry microenvironments, *Propionibacteriaceae* was the dominant family encoding for the cobamide-dependent enzymes d-ornithine aminomutase, methylmalonyl-CoA mutase, and ribonucleotide reductase class II (Fig. 2). In contrast, across the remaining cobamide-dependent enzymes, hits were assigned to diverse taxa from the four major phyla on the skin (*Actinobacteria*, *Firmicutes*, *Proteobacteria*, and *Bacteroidetes*) (46). Cobamide-dependent enzymes involved in primary metabolism, including methionine synthase, epoxyqueosine reductase, ribonucleotide reductase, and ethanolamine lyase, were the most common cobamide-dependent enzymes in the data set (Fig. S2C). Notably, only 1% of species profiled have the genetic capacity to appreciably contribute to *de novo* cobamide biosynthesis (*n* = 18 species), yet approximately 39% of species encode cobamide-dependent enzymes (*n* = 638 species encoding at least one cobamide-dependent enzyme) (Fig. S2C), suggesting that the ability to carry out cobamide biosynthesis is a rather rare function on the skin. While the true number of *de novo* cobamide producers may be underestimated due to filtering of rare and singleton hits prior to analysis, these species, many of which are prevalent and abundant members of the skin microbiome, represent the core cobamide producers found on the skin. Overall, these results support a model that predicts cobamide sharing may be occurring in the skin microbiome, where a much larger number of skin taxa encode cobamide-dependent enzymes than can produce the cofactor *de novo*.

### Regulation of cobamide biosynthesis is species specific.

**FIG 3:**
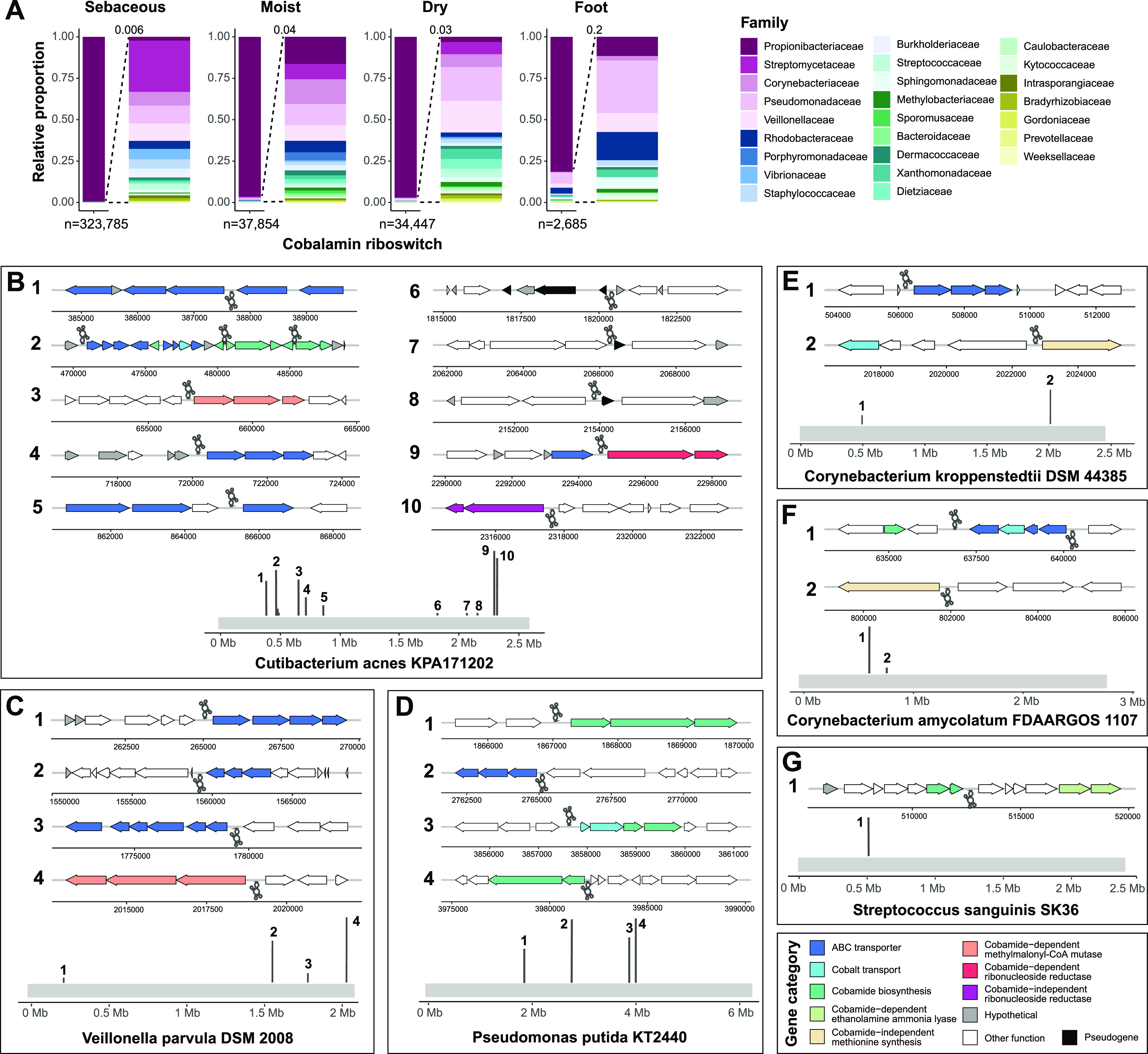
Cobalamin riboswitch regulation varies across skin taxa. (A) The taxonomic abundance of hits for cobalamin riboswitches (Rfam clan CL00101) are shown, with an expanded view of low abundance hits to the right. Total cobalamin riboswitch hits within each microenvironment are indicated. (B to G) Cobalamin riboswitch-containing reads identified from INFERNAL analysis were aligned to Cutibacterium acnes KPA171202 (B), Veillonella parvula DSM 2008 (C), Pseudomonas putida KT2440 (D), Corynebacterium kroppenstedtii DSM 44385 (E), Corynebacterium amycolatum FDAARGOS 1107 (F), and Streptococcus sanguinis SK36 (G) genomes. Dark gray lines along the light gray genome track indicate the position of mapped INFERNAL hits within the genome. Genes upstream and downstream of the riboswitches are colored by their general functional annotation. White (other function) indicates genes not currently known to be associated with cobamides. Gray (hypothetical) indicates a hypothetical protein that has no functional annotation. Right-facing gene arrows and upright dark gray riboswitch icons indicate forward strand orientation, and left-facing gene arrows and inverted riboswitch icons indicate reverse strand orientation. Genomic regions are not to scale.

Cobalamin riboswitches are cobamide-binding elements found in the untranslated region of bacterial mRNAs that regulate expression of genes or transcripts involved in cobamide-dependent metabolism, biosynthesis, and cobamide transport (47–49). Given that riboswitch regulation of cobamide-related gene expression is a widespread and well-characterized mechanism of regulation across bacteria (48), we identified cobalamin riboswitches within the metagenomes using INFERNAL (50) to further delineate cobamide usage within the skin microbiome. Consistent with our cobamide-dependent enzyme analysis, we found that diverse skin taxa encode for cobalamin riboswitches (Fig. 3A). At the species level, 94% of the riboswitch-containing reads were encoded by *C. acnes.*

To identify the pathways regulated by cobamides in *C. acnes*, we mapped sequence reads encoding cobalamin riboswitches to the *C. acnes* KPA171202 reference genome. We found that riboswitches are distributed across the genome in numerous regions and found upstream of pathways involved in ABC transport, cobalt transport, cobamide biosynthesis, and cobamide-dependent and -independent reactions (Fig. 3B). Three of the regions where cobalamin riboswitches map to in the genome (regions 6, 7, and 8) are located upstream of pseudogenes or genes of unknown function (Fig. 3B). Manual curation of these *C. acnes* KPA171202 sequences suggest that the small pseudogenes are hypothetical adhesin protein fragments and the larger downstream sequences are thrombospondin type-3 repeat containing proteins (Supplemental Material S4). Previous reports have identified differences in functional adhesion protein genes among *C. acnes* strains and have suggested that they play a role in antigenic variation and interaction with the immune system (51, 52). The role of cobalamin riboswitches in regulation of these genes is unknown but suggests that cobalamin riboswitches are regulating diverse functions that may be important on the skin, some of which are likely yet to be discovered.

The remaining riboswitch-containing reads identified were encoded by species from genera including *Cutibacterium*, *Propionibacterium*, Pseudomonas, *Veillonella*, Streptococcus, and *Corynebacterium.* Compared to *C. acnes*, genomes of select species from these genera encode relatively fewer cobalamin riboswitches. Riboswitches were located near gene neighborhoods with functions involved in cobamide biosynthesis, ABC transport, cobalt transport, and both cobamide-dependent and cobamide-independent isozymes (Fig. 3C to G). Unlike *C. acnes*, which tightly regulates cobamide biosynthesis (53, 54), our data demonstrate that riboswitches do not regulate cobamide biosynthesis in *V. parvula*, *C. kroppenstedtii*, *or C. amycolatum*. Because cobalamin riboswitches are the predominant mechanism through which cobamide metabolism is regulated (55), these findings suggest constitutive *de novo* production of the molecule by these species.

### Skin commensal Corynebacterium amycolatum produces high levels of cobamides and constitutively expresses biosynthesis genes.

**FIG 4:**
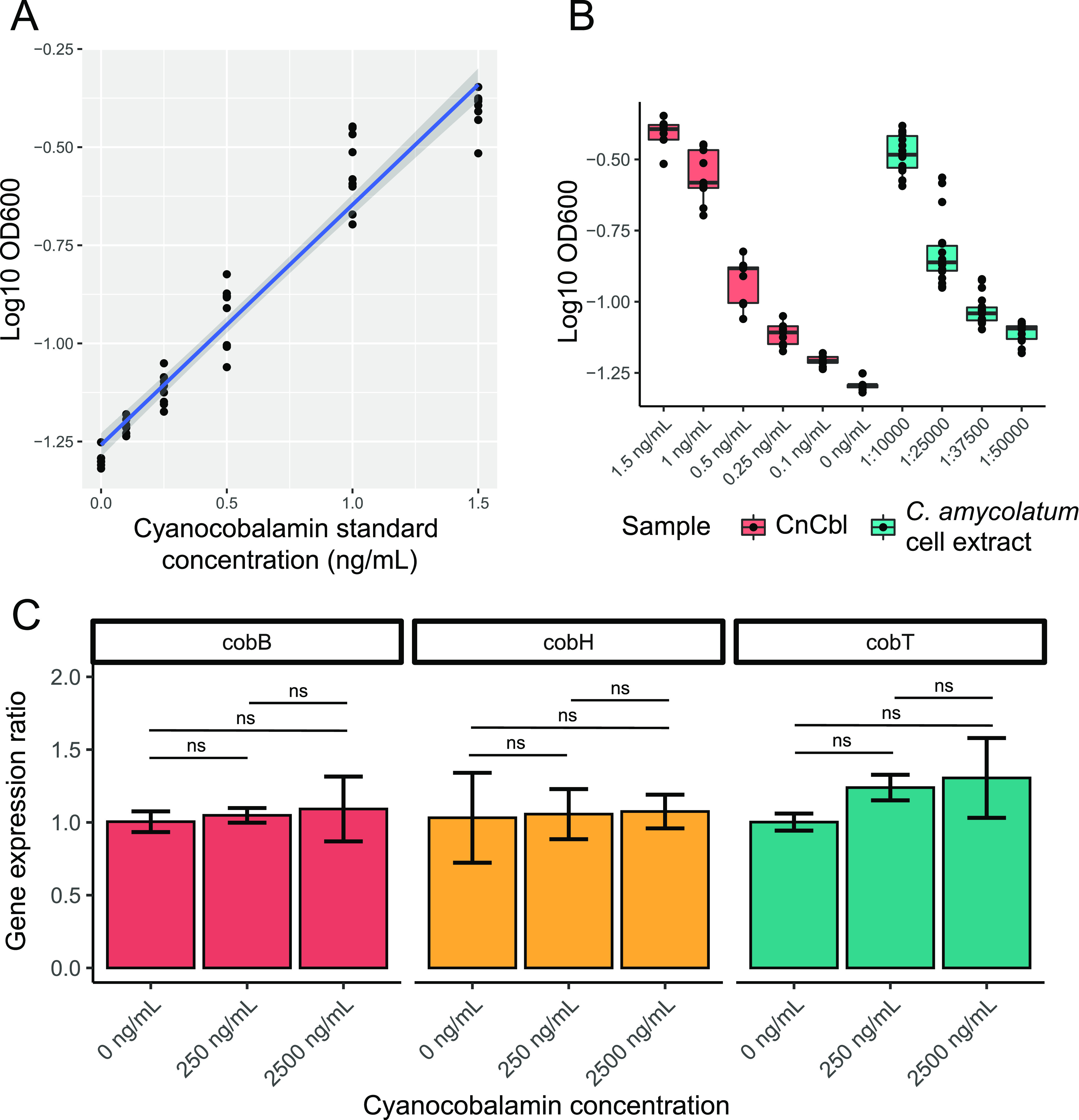
Corynebacterium amycolatum produces cobamides and constitutively expresses cobamide biosynthesis genes. (A) E. coli ATCC 14169 was used as a microbiological indicator for the detection of cobamide concentration in Corynebacterium amycolatum LK19 cell extracts. Growth of E. coli was measured in minimal media with cyanocobalamin standards between 0.1 and 1.5 ng/ml to generate a standard curve. (B) E. coli growth with cyanocobalamin standards or different dilutions of *C. amycolatum* LK19 cell extract diluted between 10,000- and 50,000-fold. OD_600_ values from 6 biological replicates and at least 3 technical replicates are shown. (C) The expression levels of *cobB*, *cobH*, and *cobT* were measured after 48 h of *C. amycolatum* LK19 culture in minimal media with cyanocobalamin supplementation (0, 250, and 2,500 ng/ml). Each culture/condition was grown in triplicate. Bars represent the mean gene expression ratio, calculated using the Pfaffl method, and error bars represent standard deviation. Cob gene expression was normalized to the 16S rRNA gene. Significance testing between cyanocobalamin concentrations was performed for each cob gene using the Kruskal-Wallis test, with significance defined as *P* < 0.05.

To test *in vitro* production of cobamides by *C. amycolatum*, a predicted *de novo* cobamide producer, we isolated strains of *C. amycolatum* from healthy skin, sequenced the genomes, and confirmed that this species encodes for *de novo* cobamide biosynthesis genes. We then cultured one of the strains in a minimal growth medium and prepared cell extracts from the intracellular metabolite content. We tested the cell extract in a microbiological assay using the indicator strain Escherichia coli ATCC 14169, whose growth is proportional to cobamide concentration from 0.1 to 1.5 ng/ml (Fig. 4A). When diluted 10,000- to 50,000-fold, *C. amycolatum* cell extracts yielded growth of E. coli within the linear range (Fig. 4B), with an average cobamide amount of 1.51 ± 0.135 μg per gram of wet cell weight and an average intracellular concentration of 11.3 ± 2.37 μM. Physiological requirements of cobamides range from nanomolar to even picomolar concentrations (20). Furthermore, we show that unlike *C. acnes* (53, 54), *C. amycolatum* exhibits constitutive gene expression of the cobamide biosynthesis genes *cobB*, *cobH*, and *cobT*, located in three distinct operons, when cultured in minimal media with or without supplemented cobalamin (Fig. 4C). Overall, these results provide early evidence of *Corynebacterium* species supplying cobamides to other species on the skin.

### Cobamide producers and users shape microbial network structures.

Having determined that cobamide producers, precursor salvagers, and users are prevalent within the skin microbiome, we sought out to determine how these members may be interacting, both with each other and with members who neither use nor produce cobamides. We utilized the SPIEC-EASI statistical method to infer microbial associations between common species on the skin (see Supplemental Material S5). To generate a final consensus network for each skin microenvironment, associations had to be present in at least two of the three metagenomic studies. These associations thus represent associations between skin microbiome members that are found across individuals, across body sites within a given microenvironment, and across data sets.

**FIG 5:**
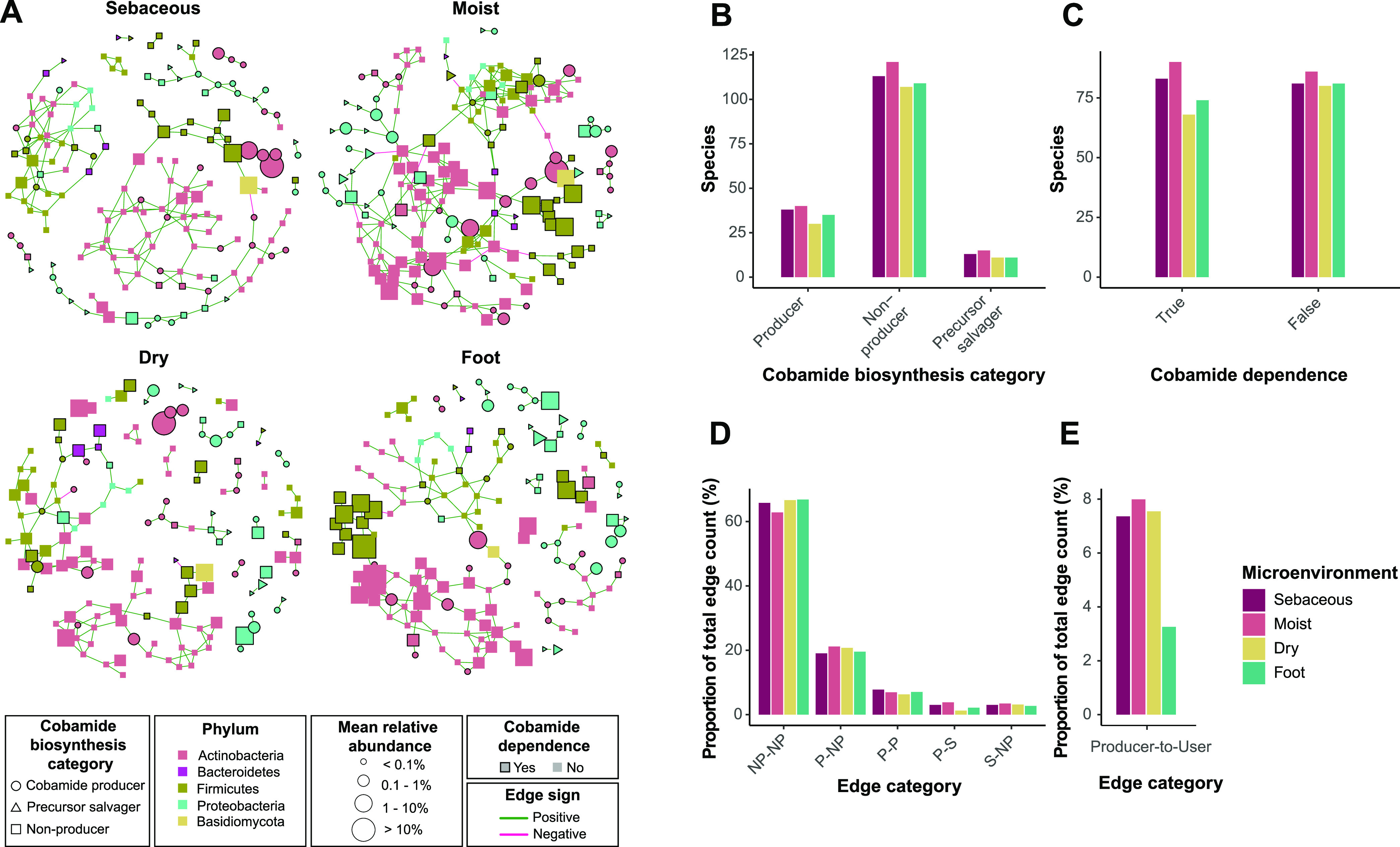
Skin microbiome networks reveal microbial associations among cobamide producers, precursor salvagers, and users. (A) The SPIEC-EASI method was used to identify microbial associations within each microenvironment of three independent skin microbiome data sets. Consensus networks are shown, representing associations identified in at least 2 of the 3 data sets. Species are represented by nodes and colored by phylum. Green and pink edges represent positive and negative associations, respectively. Node shape represents cobamide biosynthesis category and node size reflects mean species relative abundance within each microenvironment. Cobamide-dependent species are outlined in black. (B to E) In each final network, the number of species classified to each cobamide biosynthesis category (B), the number of species that are cobamide dependent or independent (C), the percentage of total edges that fall into each cobamide biosynthesis edge category (D), and the percentage of total edges that exist between cobamide producers and cobamide-dependent species that are nonproducers or precursor salvagers (E) are shown. NP, nonproducer; P, producer; S, precursor salvager.

Across microenvironments, the majority of associations are positive (Fig. 5A). The following measurements were used to quantify each network: node degree, density, transitivity, modularity, and phylum assortativity (see Table S2 for a description of these properties). The moist environment network was the least sparse and modular and the most dense and transitive, suggestive of a more interconnected community (Table S2; Fig. S3). The dry network was the most sparse and modular and the least dense and transitive, suggesting the existence of interaction modules with dense connections between species of the same module. Sebaceous and foot networks fell in the middle of this spectrum. Across all microenvironments, we observed high assortativity by phylum, indicating a preference for species to associate with other species in the same phylum.

10.1128/msystems.00677-22.3 FIG S3 For each microenvironment network, the relative frequency of nodes with each given degree is shown. Download FIG S3, PDF file, 0.01 MB. Copyright © 2022 Swaney et al. 2022 Swaney et al. https://creativecommons.org/licenses/by/4.0/ This content is distributed under the terms of the Creative Commons Attribution 4.0 International license.

10.1128/msystems.00677-22.7 TABLE S1 Description of samples used for metagenomic analysis Table S1, PDF file, 0.04 MB. Copyright © 2022 Swaney et al. 2022 Swaney et al. https://creativecommons.org/licenses/by/4.0/ This content is distributed under the terms of the Creative Commons Attribution 4.0 International license.

10.1128/msystems.00677-22.8 TABLE S2 Properties of skin microenvironment networks. Download Table S2, PDF file, 0.02 MB. Copyright © 2022 Swaney et al. 2022 Swaney et al. https://creativecommons.org/licenses/by/4.0/ This content is distributed under the terms of the Creative Commons Attribution 4.0 International license.

Across microenvironments, the distribution of species identified to be cobamide producers, precursor salvagers, and nonproducers was relatively consistent, with more nonproducers than producers or precursor salvagers (Fig. 5B). The distribution of species identified to encode cobamide-dependent enzymes (defined here as cobamide dependent) or not was also consistent across microenvironments, with a generally equal number of cobamide-dependent species to cobamide-independent species in each network (Fig. 5C). Edges were quantified based on cobamide biosynthesis category, showing more nonproducer to nonproducer edges, followed by producer to nonproducer, producer to producer, producer to precursor salvager, and finally, precursor salvager to nonproducer (Fig. 5D). The moist microenvironment has the highest number of edges between *de novo* producers and cobamide-dependent nonproducers or salvagers, followed by sebaceous, dry, and foot microenvironments (Fig. 5E). Overall, we find that associations between cobamide producers, precursor salvagers, nonproducers, users, and nonusers are distributed throughout the networks, suggesting that predicted cobamide sharing between users and producers can affect microbial interactions within the whole community. In a complex microbial community such as the skin microbiome, cobamide sharing is likely complicated and influenced by a multitude of additional direct and indirect interactions occurring within the network.

### Microbial diversity and community structure is associated with cobamide producer abundance.

Comparative genomics analysis of 11,000 genomes across the kingdom bacteria by Shelton et al. (21) revealed that a majority of bacteria are predicted to encode at least one cobamide-dependent enzyme, while only 37% of species are predicted to produce the cofactor *de novo*. Therefore, within microbial communities, cobamide sharing exists as a means to fulfill this nutritional requirement and is hypothesized to mediate community dynamics. On the skin, we find that only 1% of species encode for *de novo* biosynthesis. Two of the top most abundant genera in the skin microbiome are *Cutibacterium* and *Corynebacterium*, both of which we found to include species that are *de novo* cobamide producers. Therefore, we hypothesize that changes at the community level are associated with the presence of these cobamide-producing species. To assess this, we explored the relationship between microbiome diversity and *Cutibacterium* species that produce cobamides, as they represent the most prevalent *de novo* cobamide producers. We did not observe associations between the abundance of cobamide-producing cutibacteria and overall microbiome diversity. Rather, samples with the highest *Cutibacterium* relative abundances within each microenvironment were often the least diverse (Fig. S4A). This is likely due to a multitude of factors, including the ability of the *Cutibacterium* species to alter the local skin environment through production of antimicrobial molecules (15, 56, 57). We predict that tight regulation of cobamide production by *Cutibacterium* species (53, 54) may also contribute to this observed trend of low diversity in sites with high *Cutibacterium* relative abundance.

10.1128/msystems.00677-22.4 FIG S4 (A) Within each metagenome, the cumulative relative abundance of cobamide-producing cutibacteria was calculated. NMDS plots based on Bray-Curtis indices for healthy adult samples within each skin microenvironment are shown. Points are colored by log 10 *Cutibacterium* cobamide producer abundance and sized by alpha diversity (Shannon). (B) For each metagenome, the cumulative relative abundance of cobamide-producing *Corynebacterium* species was calculated and plotted against the cumulative relative abundance of noncobamide-producing *Corynebacterium* species. Spearman correlation analysis was performed for each microenvironment, with the correlation coefficient and *P* value listed. (C) The first (0.05%) and third (0.75%) quartiles of the cobamide-producing corynebacteria (CPC) relative abundance across all samples were used to group samples below 0.05% (Low) or above 0.75% (High) CPC abundance. Relative abundances for samples within each group are shown. Species shown are those present at greater than 10% relative abundance in at least one sample; remaining species are grouped into “Other.” Species names that are not yet validly published under the ICNP are indicated in parentheses. Download FIG S4, PDF file, 2.0 MB. Copyright © 2022 Swaney et al. 2022 Swaney et al. https://creativecommons.org/licenses/by/4.0/ This content is distributed under the terms of the Creative Commons Attribution 4.0 International license.

**FIG 6:**
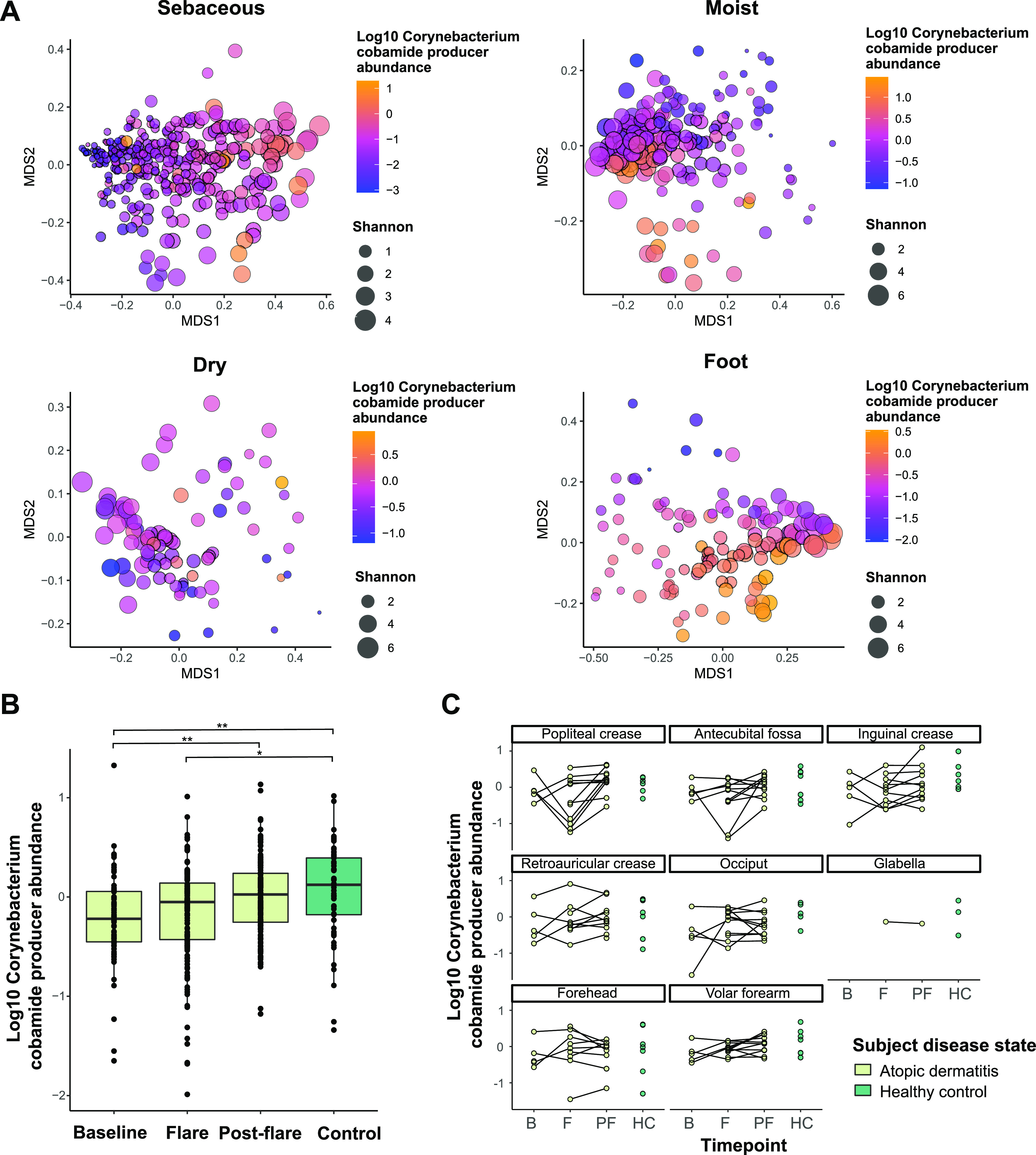
Cobamide-producing *Corynebacterium* abundance is associated with microbiome diversity and atopic dermatitis disease state. Within each metagenome, the cumulative relative abundance of cobamide-producing corynebacteria (CPC) was calculated. (A) NMDS plots based on Bray-Curtis indices for healthy adult samples within each skin microenvironment are shown. Points are colored by *Corynebacterium* cobamide producer relative abundance and sized by alpha diversity (Shannon). (B) The relative abundance of CPC in pediatric atopic dermatitis patient samples at baseline, flare, and postflare time points or in healthy control subjects. A Kruskal-Wallis test was performed followed by Dunn’s test with Bonferroni correction to determine statistical significance between groups (*, *P* < 0.05; **, *P* < 0.01). The sample size for each group are as follows: baseline *n* = 70, flare *n* = 144, postflare *n* = 144, control *n* = 57. (C) The relative abundance of CPC in each individual skin site sampled. Black lines connect time points for a given patient. Certain sites were sampled from both sides of the body, therefore each point represents the average abundance of for each individual at the specified skin site.

In contrast to *C. acnes* regulation of cobamide biosynthesis, we found that cobamide-producing corynebacteria (CPC) constitutively express cobamide biosynthesis genes (Fig. 4C). Thus, we evaluated CPC abundance and overall microbiome diversity using nonmetric multidimensional scaling (NMDS) ordination of Bray-Curtis dissimilarity indices of the dry, foot, moist, and sebaceous sites. Our analysis reveals grouping of samples that follows increasing gradients of both alpha diversity and CPC abundance, where alpha diversity increases as CPC abundance increases (Fig. 6A; Supplemental Material S9). This was most apparent for samples from sebaceous, moist, and foot sites. However, in the sebaceous and moist microenvironments, we also observed a positive correlation between non-CPC abundance and diversity (Supplemental Material S9), which we attribute to the finding that CPC and non-CPC are correlated with each other (Fig. S4B). We hypothesize that in addition to providing the vitamin directly to dependent species, cobamide sharing may also affect community members indirectly via metabolite production arising from cobamide-dependent reactions, which could explain the observation that non-CPC correlate with CPC. Furthermore, communities with a low CPC abundance are usually dominated by Cutibacterium acnes, whereas communities with high CPC abundance show an expansion of other skin taxa inside and outside the *Corynebacterium* genus and an overall more even species distribution within the community (Fig. S4C).

### Cobamide producers are depleted in atopic dermatitis.

A decrease in microbiome diversity is associated with increased pathogen colonization in dermatological disease, including atopic dermatitis (AD) (58, 59). To assess the potential role of CPC in the AD skin microbiome, we analyzed 417 metagenomes from a cohort of 11 pediatric AD patients and 7 healthy controls (8). Microbiome structures exhibited a higher level of variability compared to the adult cohorts, with weak grouping of samples based on alpha diversity or CPC abundance (Fig. S5). A subset of samples collected from moist sites during a flare formed a distinct group exhibiting low CPC abundance and alpha diversity. AD skin symptoms often present in moist sites such as the antecubital fossa (bend of the elbow) and popliteal fossa (bend of the knee), suggesting a relationship between microbiome structure, diversity, and CPC abundance during AD flares. Consistent with this hypothesis, we observed that CPC abundance is significantly reduced in AD patients at baseline (*P* = 0.0008) as well as during flares (*P* = 0.015) compared to healthy controls (Fig. 6B). Within individual patients, a decrease in CPC abundance between baseline and flare occurs in a subset of patients, particularly in the antecubital fossa and popliteal fossa (Fig. 6C) but the trend is not consistent. The physical properties of the skin change during AD flares, which may directly contribute to changes in microbiome diversity; however, this relationship remains unclear. Overall, differential CPC abundance is detected between disease states, suggesting there may be a relationship between cobamide production by these members and microbial community structure in atopic dermatitis, warranting further investigation.

10.1128/msystems.00677-22.5 FIG S5 Within each AD metagenome, the cumulative relative abundance of cobamide-producing corynebacteria (CPC) was calculated. NMDS plots based on Bray-Curtis indices for pediatric AD samples within each skin microenvironment are shown. Points are colored by log 10 *Corynebacterium* cobamide producer relative abundance and sized by alpha diversity (Shannon). Shapes represent disease time point or healthy control. Download FIG S5, PDF file, 0.2 MB. Copyright © 2022 Swaney et al. 2022 Swaney et al. https://creativecommons.org/licenses/by/4.0/ This content is distributed under the terms of the Creative Commons Attribution 4.0 International license.

### Cobamide biosynthesis is enriched in host-associated *Corynebacterium* species.

**FIG 7:**
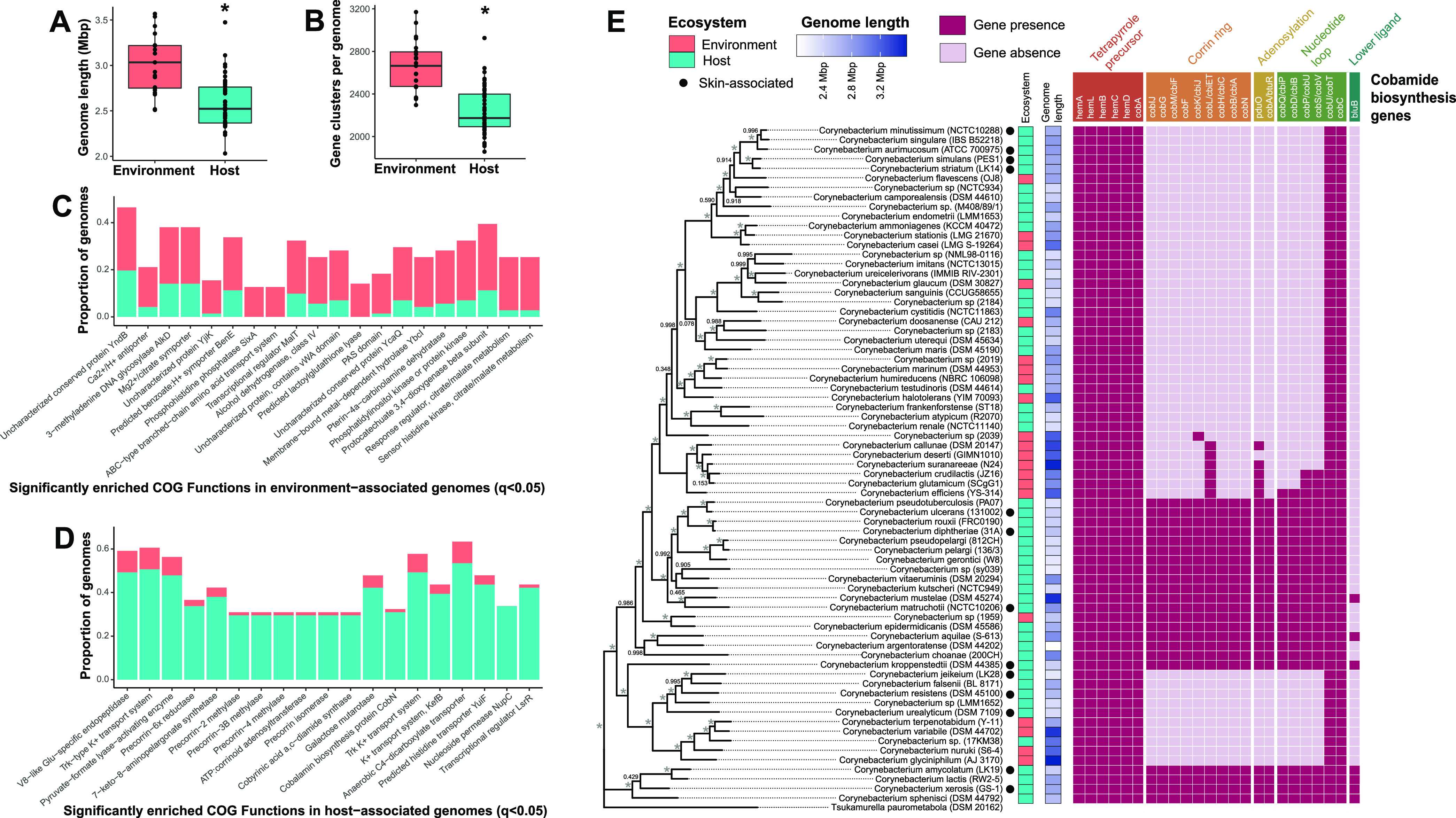
*De novo* cobamide biosynthesis is host-associated within the *Corynebacterium* genus. (A and B) Genome length (A) and number of gene clusters (B) for 71 *Corynebacterium* genomes from host- or environment-associated species were determined using anvi’o. (C and D) Significantly enriched COG functions in environment-associated (C) or host-associated (D) genomes were identified with anvi’o function “anvi-get-enriched-functions-per-pan-group.” The top 20 significantly enriched COG functions (q < 0.05) are shown, ordered by ascending significance. (E) A *Corynebacterium* phylogenetic tree based on comparison of 71 conserved single-copy genes was generated using FastTree within the anvi’o environment. The tree is rooted with Tsukamurella paurometabola, and bootstrapping values are indicated (*, 100% bootstrap support). Species are colored by host or environment association, and by genome length. KOfamScan was used to identify the presence (dark pink) or absence (light pink) of cobamide biosynthesis genes within each genome. Cobamide biosynthesis subsections are indicated and are differentially colored based on those found in Fig. 1A. Blue, host-associated; orange, environment-associated. *, *P* < 0.0001 as calculated by a two-sided Mann-Whitney U test.

Until recently, species of the *Corynebacterium* genus have been underappreciated as significant members of skin microbial communities, predominantly due to the nutritionally fastidious and slow-growing nature of many *Corynebacterium* species (46). However, sequencing efforts have revealed that corynebacteria are a dominant taxon within the microbiome, particularly in moist skin microenvironments (32, 60, 61). Our results suggest an important role for cobamide production by skin-associated *Corynebacterium* species. Because other species within the *Corynebacterium* genus occupy highly diverse habitats, including soil, cheese rinds, coral mucus, and other human and animal body sites (62), we were interested in exploring the genomic diversity within the *Corynebacterium* genus and how it relates to cobamide biosynthesis. To do so, we performed a pangenome analysis using anvi’o, which included 50 host-associated (human and animal) and 21 environment-associated (free-living) *Corynebacterium* genomes (Supplemental Material S8), acquired as complete assemblies from NCBI (*n* = 68) or as draft assemblies from human skin isolates (*n* = 3). Gene clusters (GCs), which are computed and used by anvi’o, represent one or more genes grouped together based on homology at the translated DNA sequence level (63). Across all species, 42,154 total GCs were identified. Four-hundred ninety-five of these are core GCs present in all genomes, 13,235 GCs are shared (dispensable), and 28,424 GCs are found in only one genome (species-specific/singleton) (Fig. S6). Genome size ranged from 2.0 to 3.6 Mbp, with an average of 2.7 ± 0.3 Mbp, and the number of GCs per genome ranged from 1,858 to 3,170 GCs, with an average of 2,365 ± 294 GCs (Supplemental Material S8). Host-associated species have significantly fewer GCs per genome compared to environment-associated species (2174 versus 2664, *P* value < 0.0001) and a significantly reduced median genome length (2.52 Mbp versus 3.03 Mbp, *P* value < 0.0001) (Fig. 7A and B).

10.1128/msystems.00677-22.6 FIG S6 (A) *Corynebacterium* pangenome analysis generated with anvi’o; 42,154 gene clusters (combined core, dispensable, and singletons) were identified from 71 *Corynebacterium* genomes and are ordered by gene cluster frequency (opaque, present; transparent, absent). Each gene cluster contains one or more genes contributed by one or more genomes. Genomes are colored by ecosystem association and ordered by the phylogeny based on 71 single-copy genes (unrooted). Average nucleotide identity (ANI) scale, 0.7 to 0.8. Singleton gene clusters (grey) are collapsed. (B) The number of singleton gene clusters per *Corynebacterium* genome was determined using anvi’o. (C) A two-sided Mann-Whitney U test did not reveal a significant difference in singleton gene cluster count between genomes from host- and environment-associated species. Download FIG S6, JPG file, 1.7 MB. Copyright © 2022 Swaney et al. 2022 Swaney et al. https://creativecommons.org/licenses/by/4.0/ This content is distributed under the terms of the Creative Commons Attribution 4.0 International license.

We determined functions that differ between host- and environment-associated genomes using a functional enrichment analysis (64). The top significantly enriched functions in environment-associated genomes include pathways putatively involved in amino acid transport; metabolism of various substrates, including aromatic compounds, tetrahydropterin cofactors, and citrate/malate; and other uncharacterized functions (q < 0.05) (Fig. 7C). Within host-associated genomes, we observed a significant enrichment of pathways involved in the transport of various metabolites and ions, as well as 8 Clusters of Orthologous Group (COG) functions involved in cobamide biosynthesis (q < 0.05) (Fig. 7D). To identify and validate the presence of the *de novo* biosynthesis pathway within the 71 *Corynebacterium* genomes, we scanned the genomes using KOfamScan (65). Tetrapyrrole precursor synthesis, which is shared among the cobamide, heme, and chlorophyll biosynthesis pathways (21), was conserved throughout the genus (Fig. 7E). Corrin ring and nucleotide loop synthesis was intact and conserved within 5 distinct *Corynebacterium* lineages, including those of C. diphtheriae, *C. epidermidicans*, *C. argentoratense*, *C. kroppenstedtii*, and *C. amycolatum*. The species within these groups encode for all or nearly all of the genes required for *de novo* cobamide biosynthesis, and notably, 21 out of 22 of these predicted cobamide producers are host associated. Several of the species within these lineages also were identified as core *de novo* cobamide producers within the skin metagenomes, including *C. amycolatum*, *C. matruchottii*, and *C. kroppenstedtii.* Taken together, these results demonstrate a range of cobamide biosynthetic capabilities by corynebacteria, with *de novo* producing species being almost exclusively host associated, despite a reduced genome size. Thus, we hypothesize a role for cobamides in mediating host-microbe interactions.

## DISCUSSION

Our study provides the first in depth genetic analysis of cobamide biosynthesis and use within skin microbial communities and supports the growing body of evidence that nutrient sharing is a driver of microbial community dynamics. Through analysis of skin metagenomic data, we show that phylogenetically diverse skin taxa, both high and low abundance, encode for metabolically diverse cobamide-dependent enzymes, as well as proteins involved in cobamide transport and salvage. Meanwhile, core skin species predicted to produce cobamides *de novo* (1%) are greatly outnumbered by the total number of skin species predicted to use cobamides for metabolism (39%). However, unlike other microbial communities where *de novo* cobamide producers are often minor members of the community (24–26), our results indicate that on the skin, cobamides are produced by taxa that often make up a significant proportion of the community, including *Cutibacterium* and *Corynebacterium* species (66). The significance of this is unknown but may suggest that cobamide biosynthesis is an important function for certain taxa in the skin environment. In soil microbial communities, a positive correlation has been observed between extracted DNA concentration and cobalamin concentration, suggesting that cobamides may be important in overall microbial biomass (25). On the skin, cobamide production by dominant taxa could similarly have importance in dictating community size. However, what is currently unknown is the extent to which cobamides produced by these taxa are available for community use.

Within microbial communities, cobamides are hypothesized to mediate community dynamics because of the relative paucity of cobamide producers compared to cobamide users across the bacterial domain of life (20, 21, 67). Our results suggest that on the skin, *Corynebacterium* cobamide-producing species contribute to microbiome diversity. We hypothesize that cobamide biosynthesis is one of many functions, albeit mostly unknown, that may help corynebacteria support community diversity. In addition, microbial association analysis identified associations between cobamide producers, users, and nonusers, revealing opportunities for cobamide sharing to occur and conceivably affect microbiome dynamics at a community level. Because there exists a spectrum of ecological niches on the skin, we propose that in addition to the existence of direct cobamide sharing, cobamide-mediated interactions are dependent on the spatial structure of skin microbial communities. For example, *C. acnes* is an anaerobe that predominantly resides deep within the anaerobic sebaceous follicle (68), dominating between 60 and 90% of the follicle community (69). As such, the opportunity for cobamide-mediated interactions is likely reduced as a result of the *C. acnes*-dominated sebaceous gland. Approaching the more oxygenated skin surface, the community becomes more diverse (70), which may increase the incidence of cobamide interactions and subsequent effects on community dynamics.

Furthermore, *C. acnes* and other related *Cutibacterium* and *Propionibacterium* species from the *Propionibacteriaceae* family produce cobamides with DMB as the lower ligand, requiring the *bluB* gene (71, 72). However, the bluB reaction must proceed under aerobic conditions (41). For the industrial vitamin B_12_ producer Propionibacterium freudenreichii, production of vitamin B_12_ requires both an anaerobic and aerobic growth phase, with exogenous DMB frequently added for efficient biosynthesis (73). We predict that *C. acnes* tightly regulates cobamide-related metabolism on the skin due to a preference or possible requirement for uptake and use of exogenous cobamides rather than *de novo* cobamide biosynthesis, which is limited by oxygen-requiring DMB synthesis in the anaerobic environment. To fulfill cobamide requirements when unable to synthesize the cofactor under anaerobic conditions, *C. acnes* may acquire cobamides and cobamide precursors from other species or the host (53).

Corynebacteria are well equipped for growth on the skin due to their “lipid-loving” and halotolerant nature, allowing them to thrive in moist and sebaceous skin microenvironments (74). However, many questions remain about the processes that govern skin colonization and microbe-microbe interactions by this relatively understudied skin taxa. We show that host-associated *Corynebacterium* species, including several skin commensals, encode for *de novo* cobamide biosynthesis. A key question that arises from this finding is why some host-associated *Corynebacterium* species have retained the *de novo* cobamide biosynthesis pathway, while others have not. Our results show that *Corynebacterium* species encode for cobamide-dependent methionine synthase, methylmalonyl-CoA mutase, and ethanolamine ammonia lyase, consistent with previous findings by Shelton et al. (21). Therefore, cobamides are likely produced by corynebacteria to fulfill metabolic requirements in methionine, propionate, and glycerophospholipid metabolism. Alternative cobamide-independent pathways exist for these functions; therefore, cobamides may confer a distinct advantage for these species during niche colonization on the skin. Indeed, *metE*, the cobamide-independent methionine synthase, is sensitive to oxidative stress and has reduced turnover compared to metH in E. coli (75–77). The skin in particular is subject to high oxidative stress as a result of metabolic reactions, cosmetics, and UV irradiation exposure (78–80).

Limitations of the study include the use of read-based profiling to assign gene function to specific skin taxa. While alternative methods such as metagenome assembly could be applied, we chose a read-based approach in order to provide a comprehensive and semiquantitative analysis of cobamide biosynthesis and dependence in the skin microbiome. With assembled metagenomes, there can be a bias toward abundant or easy-to-assemble species; therefore, our methods have the advantage of capturing diverse and nondominant species involved in cobamide biosynthesis and use on the skin. Finally, rigorous benchmarking of our read-profiling methods using mock community samples showed that cobamide-related genes from very low abundant species (<0.2%) within the community could be accurately detected and taxonomically classified (Fig. S1). In future studies, the use of metagenome assembly and metagenome-assembled genomes may be valuable for providing a higher resolution insight into the role of cobamides and novel pathways they may be regulating in the skin microbiome. In addition, the observed differences in microbiome structure and diversity in relation to CPC abundance in the pediatric atopic dermatitis metagenomes were not as strong as those observed in the healthy adult skin microbiome. This could be attributed to the small sample size and heterogeneity of atopic dermatitis, therefore, future investigation would benefit from a larger sample population.

The use of genomics in identifying and predicting microbial interactions is incredibly powerful, particularly for cobamides, whose biosynthesis, use, and regulation is easily identified in prokaryotic genomes. Our study further provides genomic evidence that cobamide biosynthesis is a rather rare, yet important, function across bacteria and within microbial communities (21–23, 25, 26, 37, 39, 81, 82). *In vitro* studies have corroborated the importance of cobamide-mediated interactions, providing evidence that cobamides are shared in pairwise coculture systems (83–86) and in complex communities (87) and that exogenous cobamide supplementation can induce changes in community composition (27, 88), transcriptional response (31, 89), and cobamide availability (30). Furthermore, the use of *in vivo* and mesocosm models have demonstrated that cobamide supplementation can influence microbial community dynamics (28, 29, 89) in natural microbial communities. However, the number of studies directly measuring cobamide sharing within microbial communities remain limited. This is in part due to the paucity of available models for studying complex synthetic microbial communities, particularly for the human microbiome. Additionally, cobamides are generally required in minute amounts, thus measuring picomolar concentrations of individual cobamides directly through methods such as mass spectrometry remain technically challenging. In the future, the development of models to provide experimental evidence of direct cobamide sharing between members of the skin microbiome will provide critical insights to the extent cobamides shape microbial metabolism, physiology, and community-wide interactions.

In conclusion, we have found that cobamide dependence is widespread across the phylogenetic diversity of the skin microbiome, but only a small number of skin taxa are capable of *de novo* production, including several species of the *Corynebacterium* genus. Within skin microbial communities, relative abundance of these CPC is strongly associated with richer microbial communities, suggesting that cobamides should be included in the growing list of factors serving as important mediators of microbiome structure and skin health (15, 60, 90–92). We also show that within the *Corynebacterium* genus, *de novo* cobamide biosynthesis is a host-associated function; 20 of 21 species encoding *de novo* biosynthesis are host associated. Future studies to interrogate the role of cobamides in microbe-microbe and microbe-host interactions will provide insight into the key roles that microbially derived metabolites play in microbial community dynamics and host health.

## MATERIALS AND METHODS

### Subject recruitment and sample collection.

Healthy adult volunteers were recruited from the University of Wisconsin-Madison Microbial Sciences Building in Madison, WI, USA, from July through November 2019 under an institutional review board approved protocol. The single eligibility requirement was that the subject is over 18 years of age. Subjects provided written informed consent before participation. During each visit, 8 skin sites were sampled that represent the physiologically diverse microenvironments of the skin: sebaceous (alar crease, occiput, back), moist (nare, antecubital fossa, umbilicus), dry (volar forearm), and foot (toe web space). Samples were collected by wetting a sterile foam swab (Puritan) with nuclease-free H_2_O and swabbing an approximately 1 × 1 in. area of the right lateral skin site for 15 rotations. Swabs were collected into 300 μl Lucigen MasterPure Yeast Cell Lysis solution and stored at −80°C until DNA extraction. Negative-control air swabs (*n* = 13), room swabs (*n* = 4), extraction kit controls (*n* = 2), and mock community samples (*n* = 2) were collected and prepared for sequencing as well. The mock communities used were ATCC MSA-1003 (20-strain staggered community) and ATCC MSA-1005 (6-strain even skin community).

For extraction, samples were thawed on ice and incubated shaking at 37°C for 1 h in an enzymatic cocktail of ReadyLyse (Epicenter), mutanolysin (Sigma), and lysostaphin (Sigma). Swabs were then centrifuged in a filter tube insert (Promega) for 60 s at 21,300 × *g* to remove all liquid from the swab. The liquid was added to a glass bead tube (Qiagen) and vortexed for 10 min followed by incubation at 65°C shaking for 30 min and 5 min on ice. The liquid was removed and added to MPC protein precipitation reagent, vortexed thoroughly, and centrifuged for 10 min at 21,300 × *g*. The resulting supernatant was combined with isopropyl alcohol and column purified using the Invitrogen PureLink Genomic DNA extraction kit. Finally, DNA was eluted in 50 μl elution buffer.

### Metagenomic sequencing, processing, and taxonomic classification.

Extracted DNA was prepared for sequencing by the University of Minnesota Genomics Center (UMGC) using the Nextera XT DNA Library Prep kit (Illumina). Sequencing of the libraries was performed by UMGC on a NovaSeq (Illumina 2 × 150 bp reads). We obtained 289 samples and 5.2 billion reads of nonhuman, quality-filtered, paired-end reads, with a median of 17.4 million paired-end reads per skin sample.

Quality filtering, adapter removal, human decontamination, and tandem repeat removal were performed using fastp v0.21.0 (93) and KneadData v0.8.0. Taxonomic classification and abundance estimation was performed using Kraken 2 v2.0.8-beta (35) and Bracken v2.5 (36), with a custom database that included complete bacterial, viral, archaeal, fungal, protozoan, and human genomes, along with UniVec core sequences, from RefSeq. We further modified the custom database to separate plasmid sequences from the RefSeq genomes, as we and others have observed incorrect taxonomic assignment of plasmid sequences using RefSeq taxonomy with Kraken 2 (94). Potential contaminant species were identified and removed using the prevalence method in decontam v1.10.0 (95) using extraction and air swab controls and through manual inspection of air swab samples compared to matched skin swabs, ensuring a high-quality sequence set. For each batch of samples, grouped by either sampling date or extraction date, the threshold set for decontam was between 0.3 and 0.4. The exact threshold was determined by examining the score histogram for each batch and selecting a value below the high-score mode (95). The proportion of reads identified as contaminant sequences in each sample is listed in Supplemental Material S1, which can be found on GitHub (https://github.com/Kalan-Lab/Swaney_etal_CobamidePaper).

To compare analyses across studies, an additional 906 human skin shotgun metagenomic samples from Oh et al. (32) and Hannigan et al. (33) were retrieved from the Sequence Read Archive (SRA) under BioProject IDs PRJNA46333 and PRJNA266117, respectively. Metagenomic reads across multiple SRA run accessions from the same biological sample were pooled, processed for quality control, and assigned taxonomy using the methods outlined above. In all, the metagenomic data represents the skin microbial communities across 21 distinct sites from 66 healthy individuals. Sample information is described in Supplemental Material S1 and S2.

### Choice of profile HMMs for skin metagenome survey of cobamide biosynthesis and use.

Profile HMMs, retrieved from the TIGRfam and Pfam databases, were used to detect cobamide biosynthesis, cobamide transport, and cobamide-dependent genes within skin metagenomic sequencing data. A total of 11 cobamide biosynthesis marker genes were selected because of their broad distribution throughout both the aerobic and anaerobic biosynthesis pathways and their presence within taxonomically diverse cobamide producer genomes (21, 25, 37). *cbiZ* was included as a marker of cobamide remodeling, and *btuB* was included to assess cobamide transport. Nineteen cobamide-dependent enzymes and proteins with B_12_-binding domains were chosen to evaluate cobamide use. The single-copy conserved gene *rpoB* was used as a phylogenetic marker to assess microbial community structure within each metagenome and as a proxy for sequence depth. All cobamide-associated genes used in this analysis can be found in Supplemental Material S3.

### Metagenomic sequence search using HMMER.

Sequencing reads from the present study, Oh et al., and Hannigan et al. were converted to FASTA format, retaining forward read files for analysis. For biological samples with multiple SRA run accessions, only the largest run when considering base pair count was included for analysis (Supplemental Material S1 and S2). Metagenomes were translated to each of 6 frame translations using transeq from the emboss v6.6.0 package (96). The program hmmsearch from HMMER v3.3.1 (34) was used with default parameters and an E-value cutoff of 1E-06 to scan the metagenomic sequencing reads for homology to each cobamide-related HMM. To reduce the number of spuriously mapped reads while still allowing for identification of gene-encoding sequences from organisms that may diverge over the length of the HMM profile, a gene was counted as present in a given sample if at least 20% of the gene length was covered by sequence reads. If a read aligned to multiple HMMs, the hit with the lowest E value and/or highest bit score was retained. The resulting hits were taxonomically classified to the species level using Kraken 2 (35) and Bracken (36). The number of hits for each gene was normalized to HMM length when analyzing individual metagenomes and to both HMM length and sequencing depth when analyzing groups of metagenomes. To reduce the rate of rare and singleton hits, species-gene pairings that did not appear in at least five samples from two or more data sets were excluded from further analysis. The data set was also filtered to exclude hits to the list of contaminant species identified as described above. Within individual metagenomes, the contribution of each taxon to cobamide biosynthesis was calculated by dividing the number of biosynthesis gene hits assigned to a given taxon by the total number of biosynthesis gene hits within the sample. Taxonomic frequency profiles were generated for each cobamide-related gene by dividing the normalized number of gene hits per taxon by the total normalized number of gene hits.

### Metagenomic sequence search using INFERNAL.

Covariance models (CMs) for 3 cobalamin riboswitches from the Rfam clan CL00101 were retrieved from the Rfam database (97) (Supplemental Material S3). The program cmsearch from INFERNAL v1.1.2 (50) was used with default parameters and an E-value cutoff of 1E-06 to scan the metagenomes for RNA homologs to cobalamin riboswitches. The methods following hit identification are the same as described above for HMM analysis, except that the number of hits for each riboswitch were not normalized by CM length because the read lengths and CM lengths were relatively similar.

### Mapping of cobalamin riboswitch hits to genomes.

Reads identified from INFERNAL were aligned against the complete genomes of Cutibacterium acnes KPA171202, Veillonella parvula DSM 2008, Pseudomonas putida KT2440, Corynebacterium amycolatum FDAAROS 1107, and Streptococcus sanguinis SK36 using bowtie2 v2.3.5.1 (98) and visualized in R with the ggbio v1.30.0 package (99). Genes upstream and downstream of the aligned reads within each genome were assigned functions based on NCBI RefSeq annotations and visualized using the gggenes v0.4.0 R package. Genes within genomic regions that encoded for a cobalamin riboswitch but had no genes currently known to be under cobalamin riboswitch control were assigned putative functions based on the top hit from NCBI BLAST searches against the nt/nr nucleotide collection database.

### SPIEC-EASI microbial network inference.

The statistical method SPIEC-EASI from the SpiecEasi R package v1.0.7 (100) was used to identify associations between microbial species in the skin metagenomes. Samples included for analysis are indicated in Supplemental Material S1 and S2; samples with comparatively low read counts within each data set were excluded. Species were included for analysis if they were present at greater than 0.015% average abundance and identified in at least 55% of the samples, resulting in 185 final species (Supplemental Material S5). These cutoffs were selected to attain a species number of approximately 200 to mirror that of Kurtz et al. (100), the developers of SPIEC-EASI, and to select species prevalent across individuals and data sets. SPIEC-EASI (neighborhood selection mode) was performed on samples grouped by both microenvironment and study, resulting in 12 total networks. Consensus networks were created for each microenvironment by merging sign-consistent edges from the node and edge sets identified for each study, requiring that each edge appear in at least 2 of the 3 data sets (101). The R package igraph v1.2.6 (102) was used for network visualization and calculation of topological network properties.

To incorporate cobamide biosynthesis and dependence information into the network analysis, data from Table S5 of Shelton et al. (21) were used to assess the presence of cobamide-dependent enzymes and the potential for cobamide biosynthesis or precursor salvage in each species in the consensus networks. For each species, the presence of 7 cobamide-dependent enzymes (“B12-dependent RNR,” “metH,” “methylmalonyl-CoA mutase family,” “ethanolamine ammonia lyase,” “B12-dependent glycerol/diol dehydratase,” “d-ornithine 4,5-aminomutase,” and “epoxyqueosine reductase”) was determined, and the “cobamide biosynthesis category” was used to assign cobamide biosynthesis potential. These 7 cobamide-dependent enzymes were chosen because they represent those most abundant on the skin (Fig. 3). For any consensus network species absent from the data set of Shelton et al., KEGG Ontology (KO) identifiers in Supplemental Material S6 were searched against NCBI RefSeq complete assembled genomes for each species using KOfamScan, a functional annotation program based on KOs and HMMs (65). Genomes were then scored for cobamide biosynthesis category based on the presence of certain sets of cobamide biosynthesis genes (see Supplemental Material S5 for genomes included in analysis and Supplemental Material S6 for biosynthesis scoring criteria).

### Microbiome diversity analysis of *Corynebacterium* cobamide producers.

For microbiome diversity analyses of the healthy adult skin microbiome, metagenomes from the present study and Oh et al. (32) were subsampled to 1.5 million read counts using rarefy_even_depth() from the phyloseq R package v1.34.0 (103), discarding samples below this read count cutoff. Samples included for analysis are indicated in Supplemental Material S1 and S2; samples from Hannigan et al. (33) were excluded from analysis due to comparatively lower sequencing depth: median 1.2 million (Hannigan) versus 17.4 million (this study) and 16.9 million (Oh) final paired-end reads. To adjust for study effect, adjust_batch() from the MMUPHin R package v1.5.2 (104) was used. Using taxonomic abundance information, the cumulative relative abundance of *Corynebacterium* species that encode *de novo* cobamide biosynthesis was calculated for each metagenome. Alpha diversity was determined by calculating the Shannon index using the phyloseq diversity() function. For beta diversity analysis, abundances were square root transformed to give more weight to low abundance taxa, and the Bray-Curtis dissimilarity index was calculated for samples within each skin site using vegdist() from the vegan v2.5-6 package. The indices were ordinated using nonmetric multidimensional scaling with the vegan metaMDS() program. For analysis of the pediatric atopic dermatitis microbiome, 417 metagenomes from Byrd et al. (8) were accessed from the SRA, processed, and assigned taxonomy using the described methods (Supplemental Material S7). These metagenomes represent samples from 11 pediatric AD patients (at baseline, flare, and postflare time points) and 7 healthy controls. Median sequencing depth was 2.2 million final paired-end reads. Samples MET1440, MET1441, MET1449, MET1552, and MET1563 were excluded due to insufficient sequence data after processing. Analysis of *Corynebacterium* cobamide producer abundance and alpha and beta diversity was performed as outlined above.

### *Corynebacterium* comparative genomics.

Seventy-one *Corynebacterium* isolate genomes were acquired either from the National Center for Biotechnology Information (NCBI) in August 2020 as complete assemblies or from human skin isolates as draft assemblies. Supplemental Material S8 reports accession numbers and other information for each isolate genome, including ecosystem association, which was assigned using strain metadata and species-specific literature. The pangenomics workflow from anvi’o v6.2 (http://merenlab.org/2016/11/08/pangenomics-v2/) (63, 105) was used for comparative genomics analysis. Briefly, genomes were annotated using “anvi-run-ncbi-cogs,” which assigns functions from the NCBI Clusters of Orthologous Groups (COGs) database. The *Corynebacterium* pangenome was computed using the program “anvi-pan-genome” with the flags “--minbit 0.5,” “--mcl-inflation 6,” and “--enforce-hierarchical-clustering.” Average nucleotide identity between genomes was calculated using pyani within the anvi’o environment (https://github.com/widdowquinn/pyani) (106). The program “anvi-get-enriched-functions-per-pan-group” was utilized to identify enriched COGs between host- and environment-associated genomes (64). Genome summary statistics are presented in Supplemental Material S8.

### *Corynebacterium* phylogenetic analysis.

The anvi’o phylogenomics workflow (http://merenlab.org/2017/06/07/phylogenomics/) was used to create a *Corynebacterium* phylogeny. Within the anvi’o environment, single-copy core genes (SCGs) from the curated anvi’o collection Bacteria_71 were identified within each genome using HMMER (34), and the SCG amino acid sequences were concatenated and aligned using MUSCLE (107). A phylogenetic tree was then constructed using FastTree (108) within the anvi’o environment, and Tsukamurella paurometabola DSM 20162 was included as an outgroup to root the tree. To identify cobamide biosynthesis genes within the 71 *Corynebacterium* genomes, KEGG orthology (KO) identifiers from KEGG map00860 (Supplemental Material S6) were used to create a custom profile for KOfamscan. Each genome was queried against this profile, and hits to the KOs above the predefined inclusion threshold or user-defined threshold (*cobU*/*cobT*, *cobC*, and *bluB*) were considered for further analysis. Visualization of the phylogenetic tree and cobamide biosynthesis pathway completeness was performed in R with the ggtree package v2.4.2 (109).

### *Corynebacterium* minimal M9 medium composition and preparation.

We prepared 11.28 g/liter M9 salts, 0.1 g/liter glucose, and 0.2% Tween 80 in aqueous solution and autoclaved at 121°C for 15 min. We and others have observed that autoclaving a small amount of glucose (0.1 g/liter) in the presence of other media components improves rapid and abundant growth of *Corynebacterium* species in synthetic media (110). When cooled, the following media components were added at the concentrations indicated: 0.1 mM CaCl_2_, 2 mM MgSO_4_, 50 nM CoCl_2_, 6 μM thiamine-HCl, 1.9 g/liter glucose, 100 mg/liter l-arginine, and 2 mg/liter biotin.

### Corynebacterium amycolatum cell extract preparation.

*C. amycolatum* LK19, isolated from healthy adult skin, was cultured overnight in brain heart infusion (BHI) with 0.2% Tween 80. To remove residual cobamides in the media and scale up culture conditions, cells were washed 3 times with *Corynebacterium* minimal M9 (CM9) broth, inoculated into 250 ml CM9 broth (starting optical density at 600 nm [OD_600_] = 0.1), and incubated shaking at 37°C for 24 h. Cells were again washed, inoculated into 1 liter of CM9 broth (starting OD_600_ = 0.1), and incubated shaking at 37°C for 48 h. Cells were spun down at 4,000 rpm for 20 min, wet cell weight was recorded, and 20 ml methanol per 1 g wet cell weight was added for metabolite extraction. To convert cobamides to their cyano form, 20 mg potassium cyanide was added per 1 g wet cell weight, and the cell suspension was heated at 60°C for 90 min and mixed intermittently every 20 min. Following overnight room temperature incubation, cell debris was removed, and the solvent was evaporated using a rotary evaporator. The resulting extract was desalted with a C18 Sep-Pak (Waters) cartridge. Briefly, the cell extract was suspended in 10 ml H_2_O and run through the cartridge, followed by a 20-ml H_2_O wash and elution of the cobamide-containing fraction with 3-ml methanol. The desalted extract was dried in the fume hood and resuspended in 1.1 ml H_2_O for subsequent analysis.

### Microbiological cobamide indicator assay.

E. coli strain ATCC 14169, which requires either cobamide or methionine supplementation for growth, was acquired from the NRRL Culture Collection. The strain was cultured for 6 h in BHI and washed 3 times with M9 minimal medium (11.28 g/liter M9 salts [Sigma-Aldrich], 0.1 mM CaCl_2_, 0.2 mM MgSO_4_, 1 mM thiamine-HCl, 2 g/liter glucose, and 100 mg/liter l-arginine). Cells were adjusted to an OD_600_ of approximately 0.02 in M9 minimal medium. In each well of a 96-well plate, 200 μl of cells and 2.5 μl of sample (cyanocobalamin standards or *C. amycolatum* LK19 cell extract dilutions) were added. The plate was incubated stationary at 37°C for 18 h, and OD_600_ values were recorded using a BioTek Epoch 2 Microplate Spectrophotometer. A standard curve was generated using cyanocobalamin concentrations between 0.1 and 1.5 ng/ml, and this was used to calculate cobamide concentration in the cell extracts. Intracellular concentrations were estimated assuming a cellular volume of 1 μm^3^ and 8 × 10^8^ cells/ml at an OD_600_ of 1.0 (111).

### RT-PCR analysis of cobamide biosynthesis gene expression.

*C. amycolatum* was cultured in CM9 minimal media with or without supplementation of cyanocobalamin for 48 h, and total RNA was extracted using the Qiagen RNeasy Plus minikit with enzymatic and mechanical lysis. Briefly, cell pellets were resuspended in 100 μl of an enzymatic cocktail containing 50 U/ml lysozyme, 500 U/ml mutanolysin, and 2 mg/ml proteinase K in TE buffer and incubated for 15 min with intermittent vortexing. Cells were then bead beat in 2-ml glass bead tubes (Qiagen) with the kit lysis buffer for 5 cycles of 40-s bead beating on a MP Biomedicals FastPrep-24 5G homogenizer. Tubes were centrifuged at maximum speed, and supernatants were transferred to Qiagen gDNA eliminator columns. Remaining steps were carried out according to manufacturer protocol. cDNA was synthesized from total RNA with the SuperScript IV VILO Master Mix with ezDNase Enzyme (Invitrogen) following the manufacturer’s protocol. RT-PCR was performed using the PowerUP SYBR green Master Mix (Applied Biosystems) on a QuantStudio 7 Flex real-time PCR system (Applied Biosystems) using the primers listed in Supplemental Material S10. Results were normalized to those obtained from the 16S rRNA gene using TaqMan probe Ba04230899_s1.

### Quantification and statistical analysis.

The R Statistical Package was used to generate figures and compute statistical analyses. Statistical significance was verified through the Mann-Whitney U test or the Kruskal-Wallis test followed by Dunn’s test with Bonferroni correction. Correlations between CPC abundance and Shannon diversity were calculated using the Spearman rank coefficient.

### Data availability.

Supplemental Material S1 to S10 can be found on GitHub (https://github.com/Kalan-Lab/Swaney_etal_CobamidePaper) as SupplementalMaterial.xlsx. Metagenomic sequencing data generated in the current study are publicly available in the Sequence Read Archive (SRA) under BioProject PRJNA763232. *Corynebacterium* genome assemblies are publicly available from NCBI under BioProject PRJNA395377.

### Code availability.

Code used for analyses and generating figures is available on GitHub: https://github.com/Kalan-Lab/Swaney_etal_CobamidePaper.
