# Supplementary figures and images for: Cobamide Sharing Is Predicted in the Human Skin Microbiome
Source: mSystems. 2022 Aug 15;7(5):e00677-22. doi: 10.1128/msystems.00677-22 (PMC9600381; doi:10.1128/msystems.00677-22)

**A**

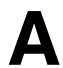

**C**

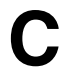

# B

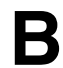

# D

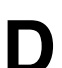

Supplement: FIG S1 [file msystems.00677-22-s0003.pdf]

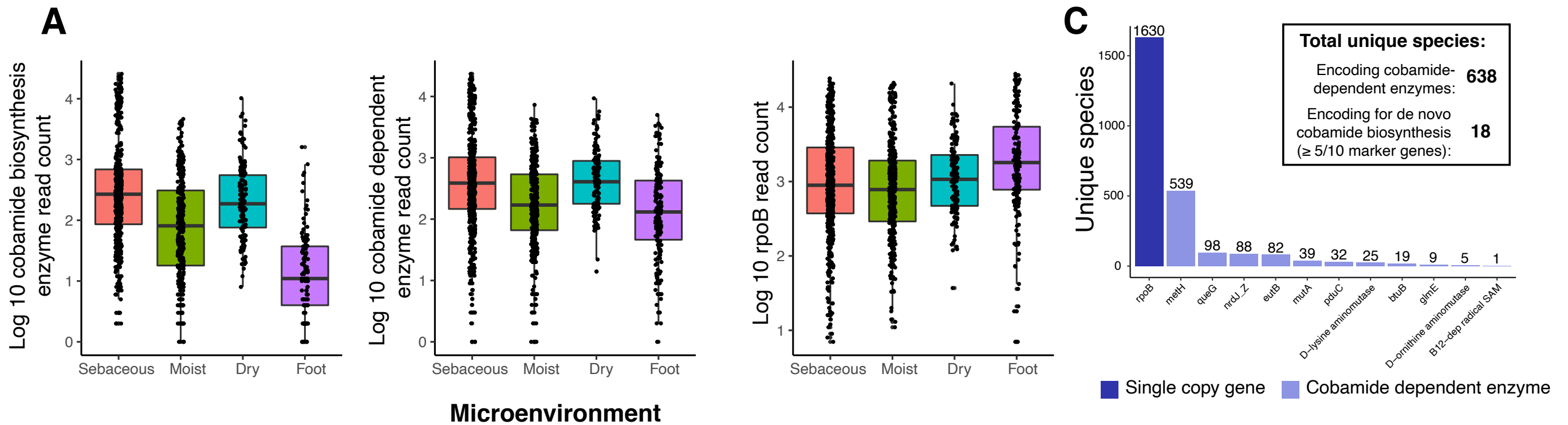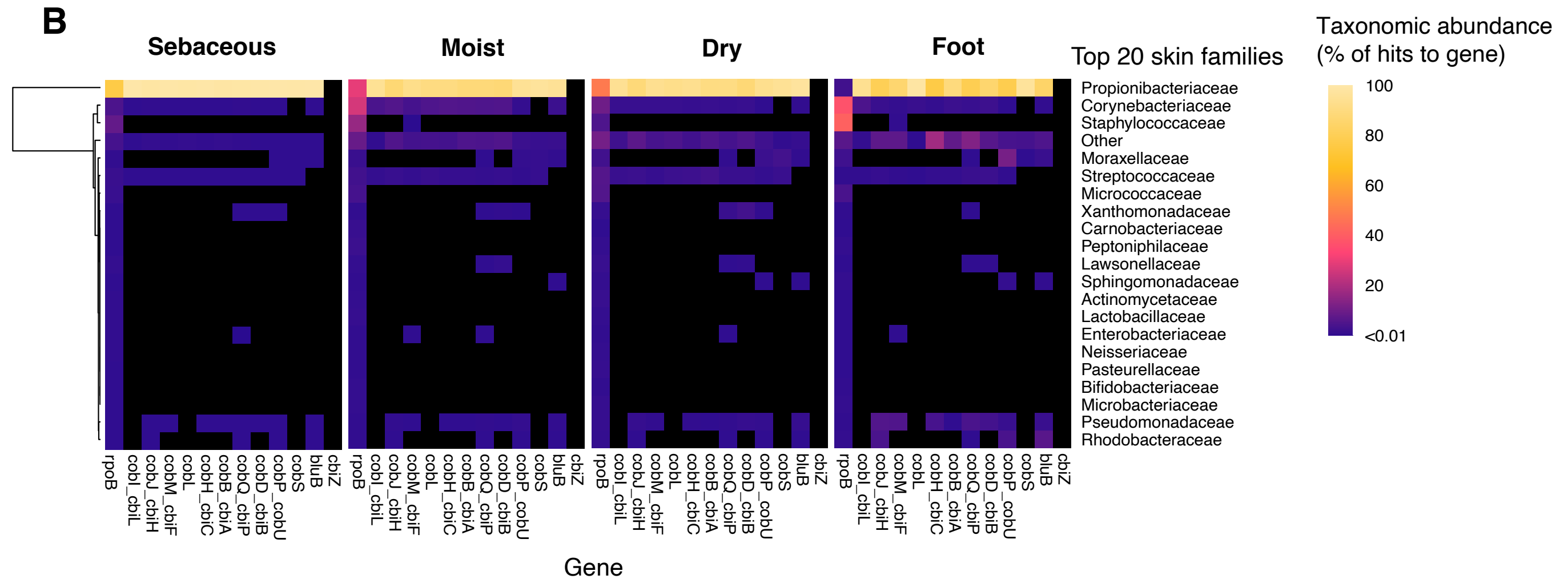

Supplement: FIG S2 [file msystems.00677-22-s0004.pdf]

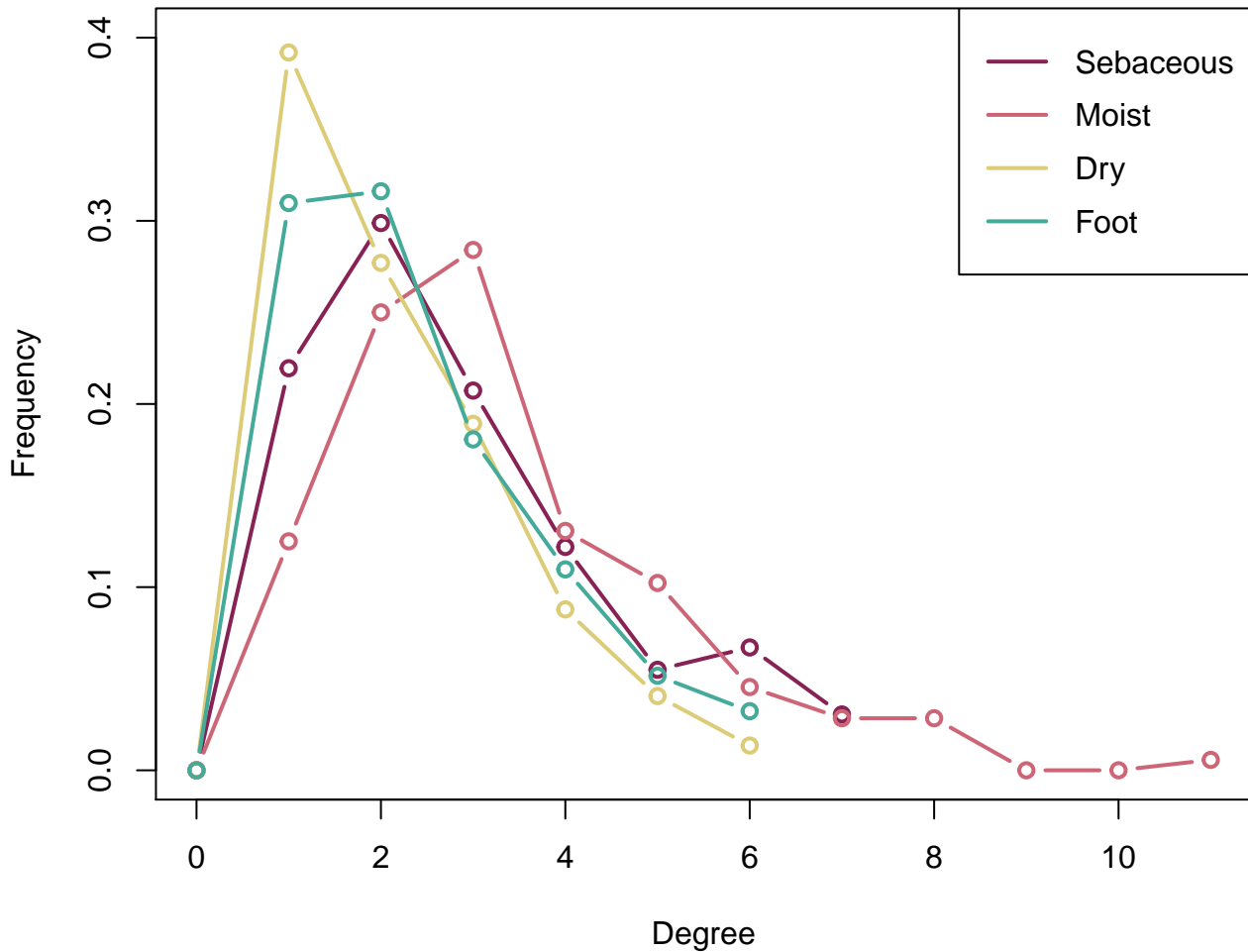

Supplement: FIG S3 [file msystems.00677-22-s0005.pdf]

A

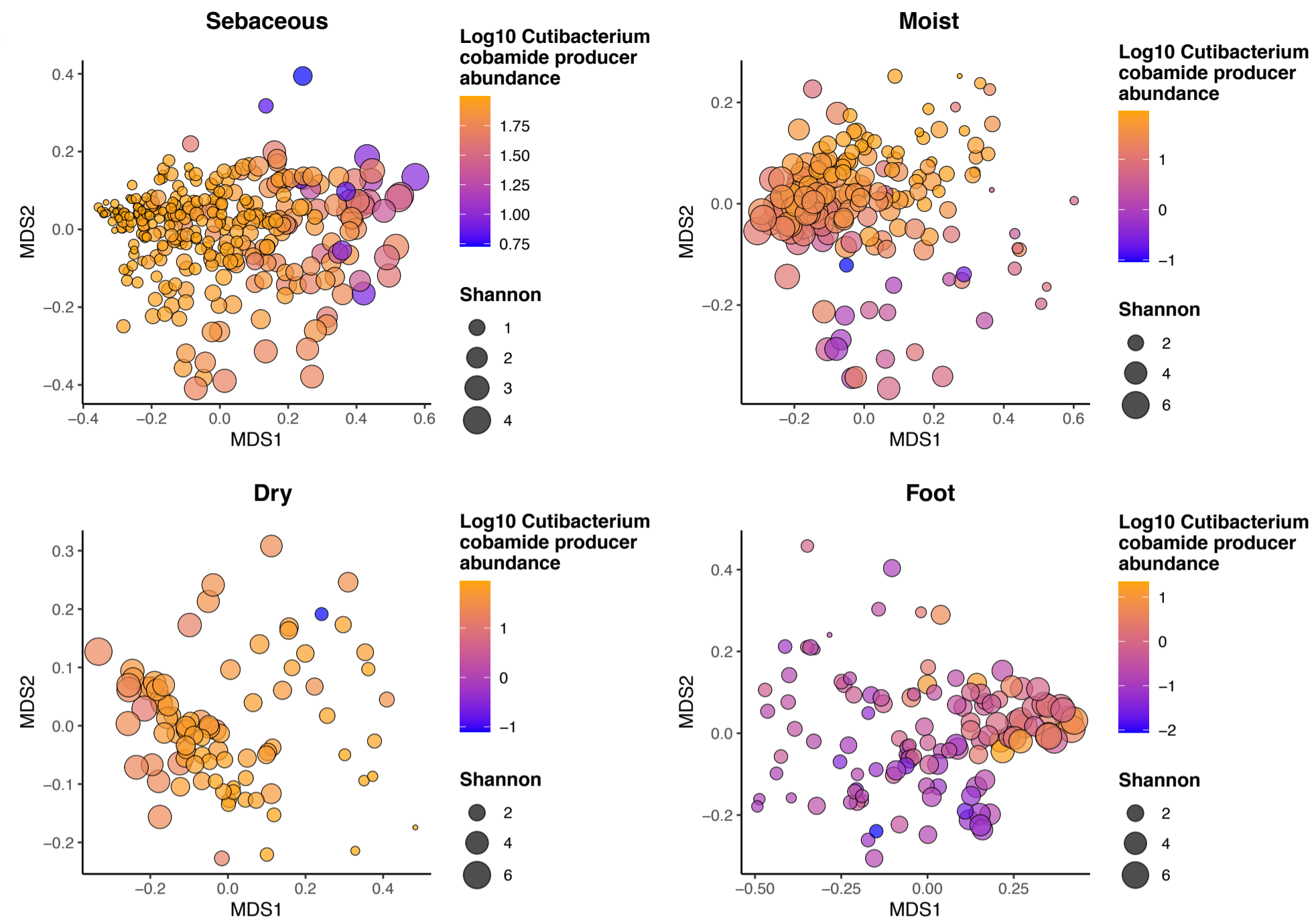

B

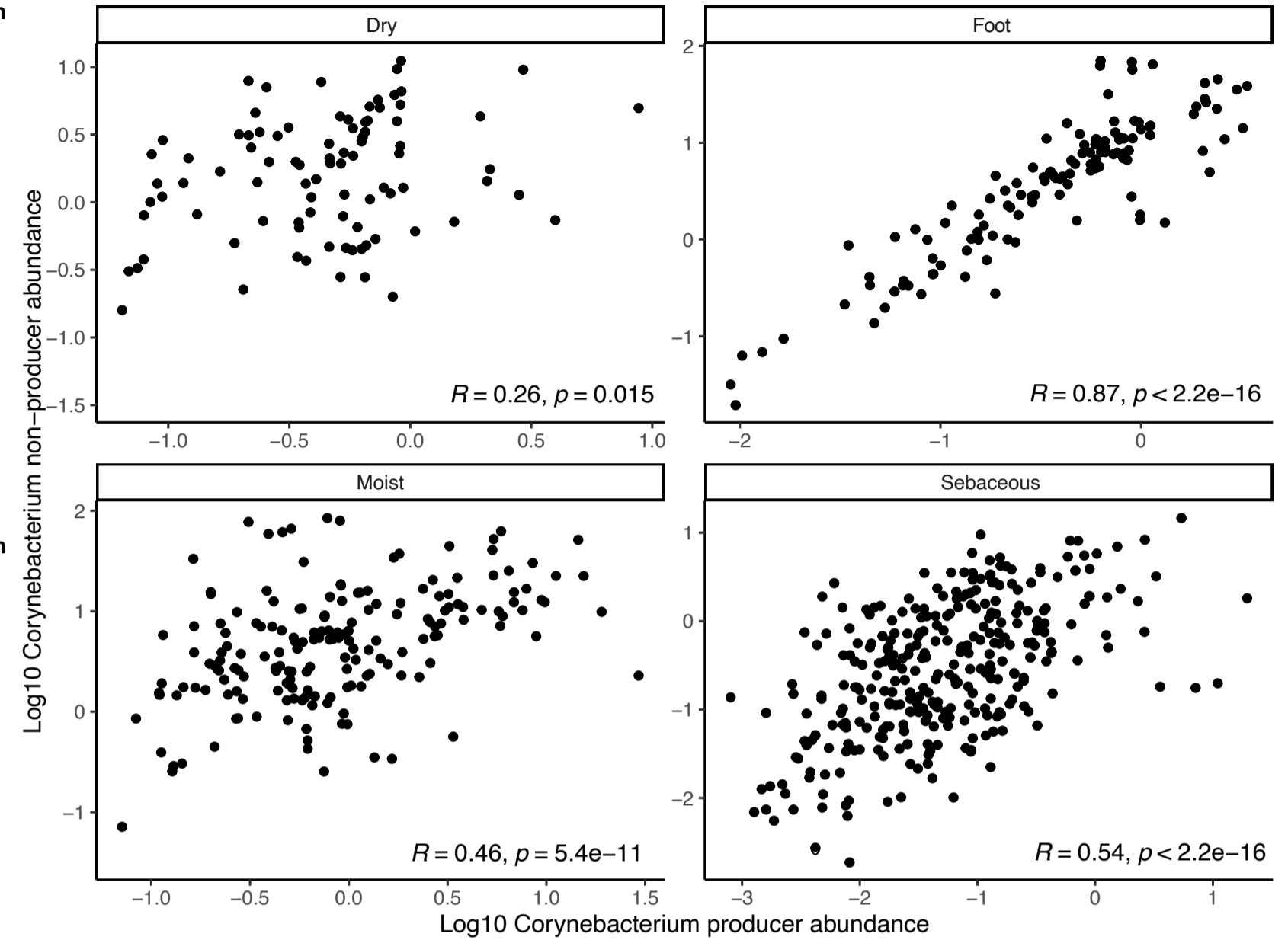

C

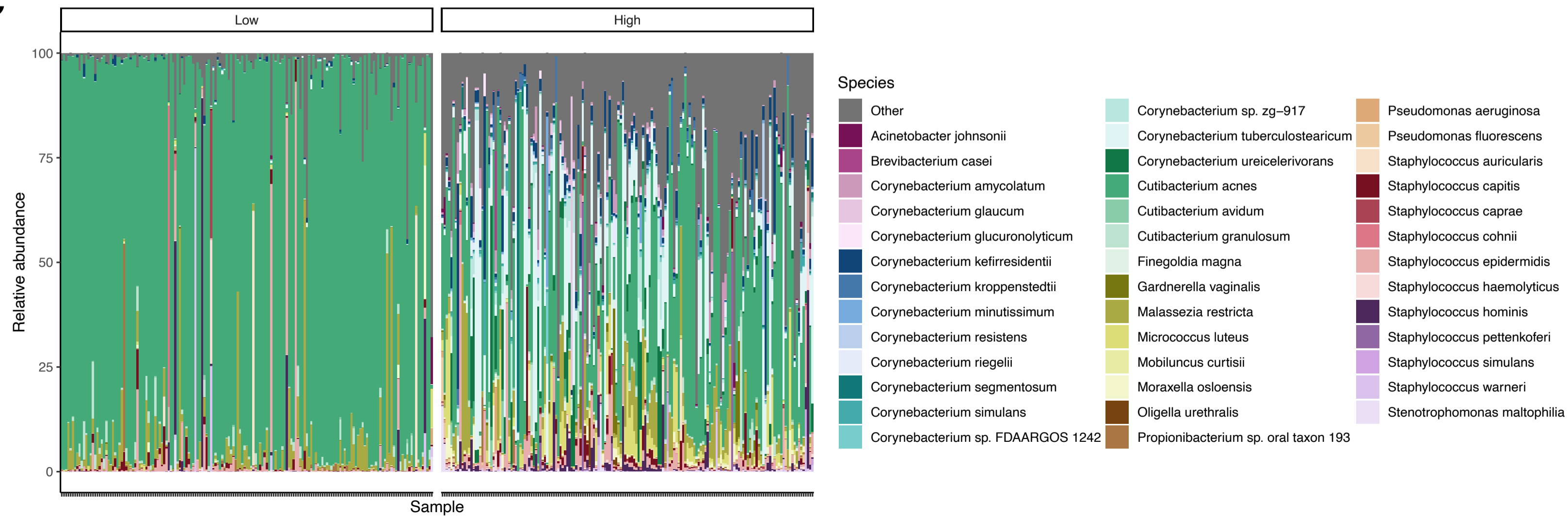

Supplement: FIG S4 [file msystems.00677-22-s0006.pdf]

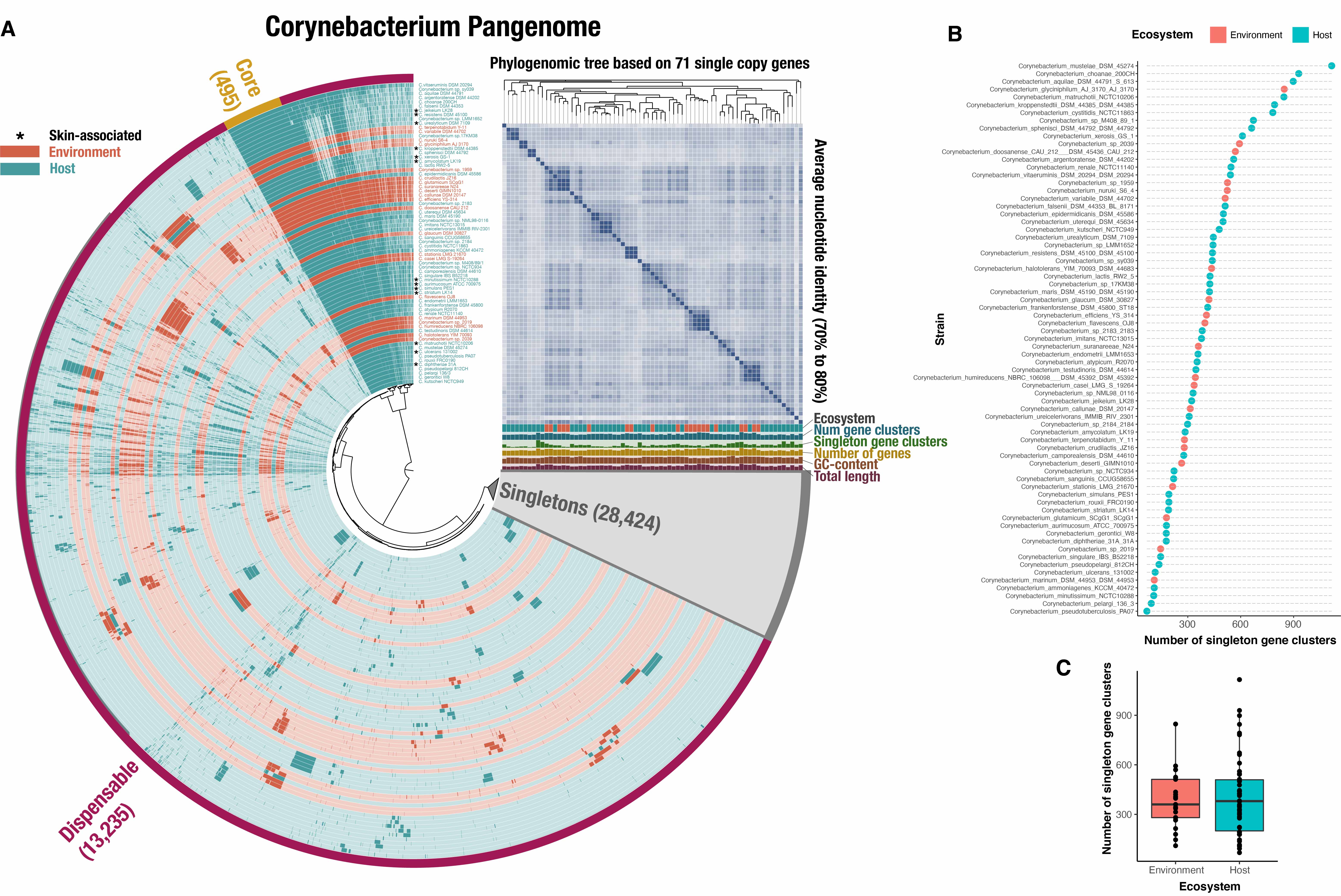

Supplement: FIG S6 [file msystems.00677-22-s0008.jpg]
