# Supplementary material for: Cobamide Sharing Is Predicted in the Human Skin Microbiome
Source: mSystems. 2022 Aug 15;7(5):e00677-22. doi: 10.1128/msystems.00677-22 (PMC9600381; doi:10.1128/msystems.00677-22)
Supplement: TABLE S1 [file msystems.00677-22-s0001.pdf]

Supplemental Table 1. Description of samples used for metagenomic analysis

**Analysis**	**Cobamide metagenome analyses**	**SPIEC-EASI analyses**	**Diversity analyses**
**Samples from present study**	**268**	**212** (low abundant samples excluded)	**212** (subsampled to 1.5 million read counts, samples below cutoff were excluded)
**Samples from Oh *et al.***	**594**	**492** (low abundant samples excluded)	**492** (subsampled to 1.5 million read counts, samples below cutoff were excluded)
**Samples from Hannigan *et al.***	**312**	**271** (low abundant samples excluded)	**0** (Sequencing depth much lower than other two studies)
**Rationale for sample exclusion**	NA	Pruning of low abundant samples performed by SPIEC-EASI developers (Kurtz, Bonneau, and Müller 2019)	Subsampling mitigates large differences in library sizes, which can impact diversity estimates (Weiss et al. 2017)
**Figure and table references**	Figures 1-3, Supplemental Figure 2	Figure 5, Supplemental Figure 3, Supplemental Table 2	Figure 6, Supplemental Figure 4, Supplemental Material S9

Supplemental References

Kurtz, Zachary D., Richard Bonneau, and Christian L. Müller. 2019. “Disentangling Microbial Associations from Hidden Environmental and Technical Factors via Latent Graphical Models.” *BioRxiv*. https://doi.org/10.1101/2019.12.21.885889.

Weiss, Sophie, Zhenjiang Zech Xu, Shyamal Peddada, Amnon Amir, Kyle Bittinger, Antonio Gonzalez, Catherine Lozupone, et al. 2017. “Normalization and Microbial Differential Abundance Strategies Depend upon Data Characteristics.” *Microbiome* 5 (1). https://doi.org/10.1186/s40168-017-0237-y.
