# Supplementary material for: Cobamide Sharing Is Predicted in the Human Skin Microbiome
Source: mSystems. 2022 Aug 15;7(5):e00677-22. doi: 10.1128/msystems.00677-22 (PMC9600381; doi:10.1128/msystems.00677-22)
Supplement: TABLE S2 [file msystems.00677-22-s0002.pdf]

Supplemental Table 2. Properties of skin microenvironment networks.

Microenvironment	Edges	Nodes	Density*	Transitivity**	Phylum Assortativity***	Modularity****	Average node degree *****
**Sebaceous**	231	164	0.017283	0.2187005	0.7036747	0.7598433	2.82
**Moist**	288	176	0.018701	0.2381974	0.7059118	0.7444059	3.27
**Dry**	159	148	0.014617	0.1843003	0.6873054	0.8310193	2.15
**Foot**	184	155	0.015417	0.2	0.8162985	0.8135633	2.37

*Density represents the number of existing relationships relative to the total possible number
** Transitivity indicates the probability that two adjacent nodes are connected and can reveal the existence of tightly connected communities
*** Phylum assortativity reflects the preference for nodes to be connected to other nodes within the same phylum
**** Modularity is a measurement of the division of a network into modules
***** Average node degree is a measure of network sparsity; a lower average node degree indicates a sparser network.
