# Supplementary material for: Cobamide Sharing Is Predicted in the Human Skin Microbiome
Source: mSystems. 2022 Aug 15;7(5):e00677-22. doi: 10.1128/msystems.00677-22 (PMC9600381; doi:10.1128/msystems.00677-22)
Supplement: FIG S5 [file msystems.00677-22-s0007.pdf]

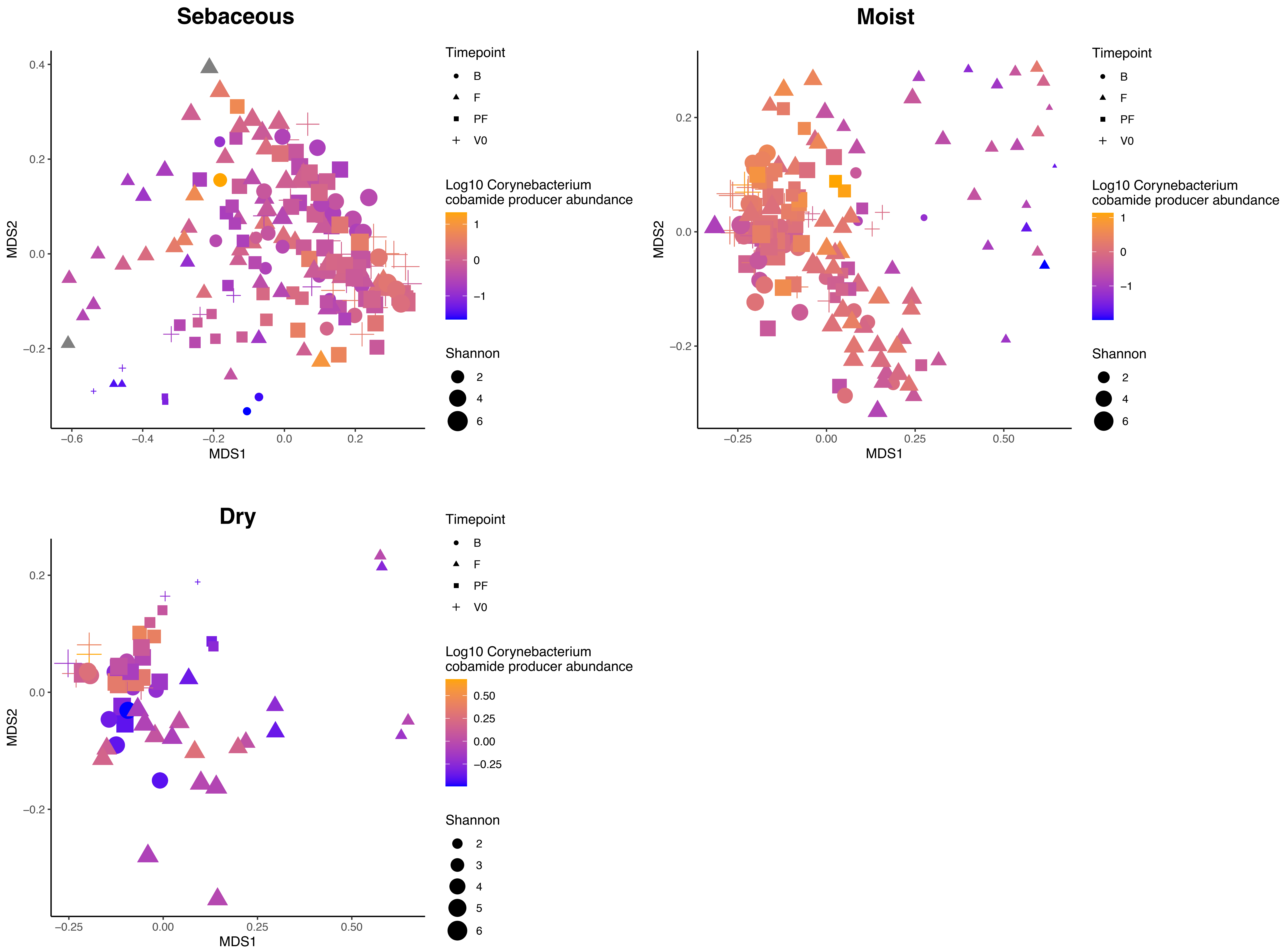
Sebaceous
MDS2
MDS1
0.4
0.2
0.0
−0.2
−0.6
−0.4
−0.2
0.0
0.2
Timepoint
B
F
PF
V0
Log10 Corynebacterium
cobamide producer abundance
1
0
−1
Shannon
2
4
6
Moist
MDS2
MDS1
0.2
0.0
−0.2
−0.25
0.00
0.25
0.50
Timepoint
B
F
PF
V0
Log10 Corynebacterium
cobamide producer abundance
1
0
−1
Shannon
2
4
6
Dry
MDS2
MDS1
0.2
0.0
−0.2
−0.25
0.00
0.25
0.50
Timepoint
B
F
PF
V0
Log10 Corynebacterium
cobamide producer abundance
0.50
0.25
0.00
−0.25
Shannon
2
3
4
5
6
